# Synthesis and Characterization of Some New Coumarins with *in Vitro* Antitumor and Antioxidant Activity and High Protective Effects against DNA Damage

**DOI:** 10.3390/molecules21020249

**Published:** 2016-02-22

**Authors:** Mounir A. I. Salem, Magda I. Marzouk, Azza M. El-Kazak

**Affiliations:** 1 Synthetic Organic Chemistry Laboratory, Chemistry Department, Faculty of Science, Ain Shams University, Abassia, Cairo 11566, Egypt; salemmai1947@yahoo.com; 2 Faculty of Education; Ain Shams University, Roxy, Cairo 11711, Egypt; az_azelkazak@hotmail.com

**Keywords:** functionalized coumarin, antitumor activity, antioxidant activity

## Abstract

Coumarins are naturally occurring oxygen heterocyclic compounds having multifarious medicinal properties, hence used as lead compounds for designing new potent analogs. The chromene butenoic acid **3** and the benzochromene butenoic acid **4** which are derived from the reaction of glyoxalic acid with 3-acetylcoumarin and 3-acetylbenzocoumarin, respectively, were reacted with different nitrogen and carbon nucleophiles to give new heterocyclic compounds. The structures of the prepared compounds were elucidated by IR, ^1^H-NMR, and mass spectroscopy. Some of the newly prepared compounds were tested *in vitro* against a panel of four human tumor cell lines namely; hepatocellular carcinoma (liver) HepG2, colon cancer HCT-116, human prostate cancer PC3, and mammary gland breast MCF-7. Also they were tested as antioxidants. Almost all of the tested compounds showed satisfactory activity.

## 1. Introduction

Coumarins are an important class of compounds of both natural and synthetic origin. Many compounds which contain the coumarin moiety exhibit useful and diverse pharmaceutical and biological activities, often depending on the substituents they bear in the parent benzopyran moiety [1,2] and, there has been a growing interest in their synthesis [3]. Some of these coumarin derivatives have been found useful in photochemotherapy, antitumor [4], anti-HIV therapy [5,6], as CNS-stimulants [7], antibacterial [8,9,10], anticoagulants [11,12,13], antifungal [14,15], antioxidant [16] agents and as dyes [17]. Natural, semi-synthetic and synthetic coumarins are useful substances in drug research [18]. Coumarins can be used not only to treat cancer, but to treat the side effects caused by radiotherapy [19,20]. Coumarin derivatives can possess not only cytostatic, but cytotoxic properties as well [21], as they can inhibit growth in human cancer cell lines [22] such as A549 (lung), ACHN (renal), H727 (lung), MCF7 (breast) and HL-60 (leukemia) and in some clinical trials they exhibited anti-proliferative activity in prostate cancer [23] malignant melanoma [24] and renal cell carcinoma [25]. Coumarin itself also exhibited cytotoxic effects against Hep2 cells (human epithelial type 2) in a dose dependent manner and showed some typical characteristics of apoptosis with loss of membrane microvilli, cytoplasmic hypervacualization and nuclear fragmentation [26].

**Figure 1:**
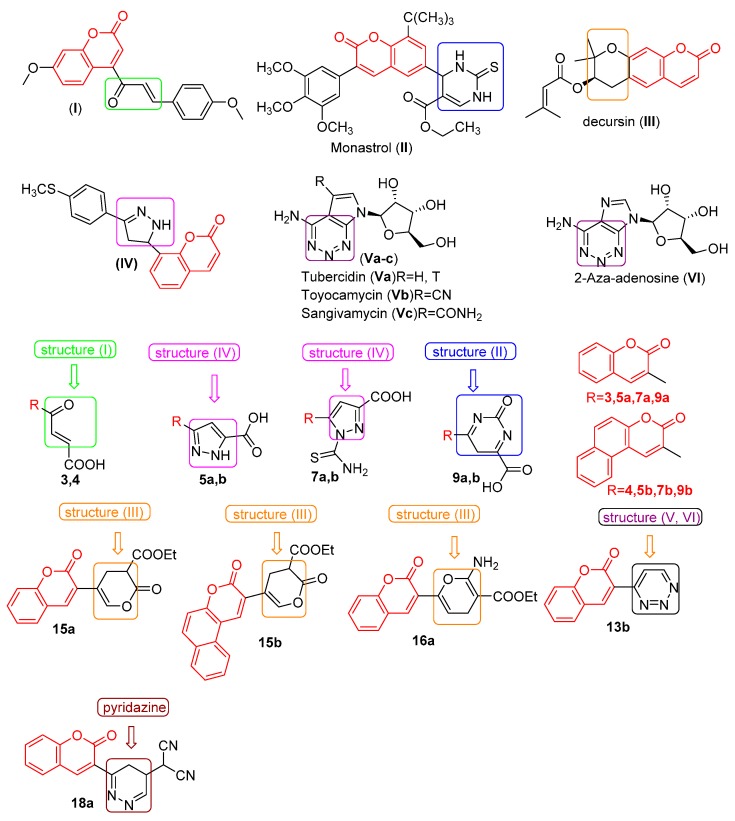
Structure of some anticancer derivatives (**I**–**VI**) and some of the designed target compounds **3**, **4**, **5a,b**, **7a,b**, **9a,b**, **13b**, **15a,b**, **16a** and **18a**.

The hormone oestrogen plays the crucial role in the development of the breast cancer, the most frequent malignant disease in women. Therefore many therapies are designed to block its activity [27]. Cinnamoyl-coumarin derivatives were especially effective in oestrogen-dependent cancers, such as breast (MCF7) and ovarian (OVCAR) cancer cell lines. These compounds are selective nonsteroidal inhibitors of 14β-hydroxysteroid dehydrogenase type 1, an enzyme that catalyzes NADPH-dependent reduction of the weak oestrogen, oestrone, into the most potent oestrogen, oestradiol [28]. Seidel *et al.* [29] synthesized a series of coumarin derivatives which carry α,β-(mono- or bis)-unsaturated ketones at the C3 or C4 position such as compound **I** (Figure 1) which strongly inhibits proliferation in the chronic myeloid leukemia K-562 and histiocytic lymphoma U-937 cell lines. This compound also inhibited the activity of histone deacetylase (HDAC), an enzyme crucial in cancer development.

Sashidhara *et al.* [27] developed new hybrid coumarin-monastrol molecules such as compound **II** (Figure 1) which showed the most potent selective activity against breast cancer cell lines MCF-7 and MDA–MB-231. This compound induced caspase-3 activation and apoptosis and caused arrest of MCF-7 cell cycle at G1 phase.

From Korean *Angelica gigas* root Kim *et al.* [30] isolated pyranocoumarin decursin **III** (Figure 1), which inhibited proliferation in bladder cancer 235J cells and also in colon cancer HCT-1116 cells. Decursin induced apoptosis in both cancer cell lines through expression of Bax protein and reduced Bcl-2 protein levels. Amin *et al.* [31] synthesized coumarins **IV** attached to pyrazoline rings (Figure 1) that have anticancer activity against the HepG2 cell line.

Drugs containing 1,2,3-triazine rings [32] originated from natural and synthetic sources are exemplified by tubercidin (**Va**), toyocamycin (**Vb**) and sangivamycin (**Vc**) (Figure 1), which have significant pharmacological activities. Tubercidin (**Va**) and its 5-substituted derivatives inhibit both DNA and RNA viruses at the concentrations that inhibit DNA, RNA and protein synthesis in mice and human cell lines. Toyocamycin (**Vb**) is a known antineoplastic antibiotic with specific antitumor activity. Sangivamycin (**Vc**) is active against L1210 leukemia, P338 leukemia and Lewis lung carcinoma and under clinical trials against colon cancer, gall bladder cancer and acute myelogenous leukemia in humans. 2-Azaadenosine **VI** (Figure 1) exhibits five times greater cytotoxicity than 8-azapurine against human epidermis carcinoma cells *in vitro*.

In continuation of our previous investigations [33,34,35,36,37], some heterocyclic compounds bearing the coumarin moiety were utilized herein for the synthesis of valuable heterocyclic ring systems having satisfactory antitumor and antioxidant activities.

## 2. Results and Discussion

### 2.1. Chemistry

**Scheme 1:**
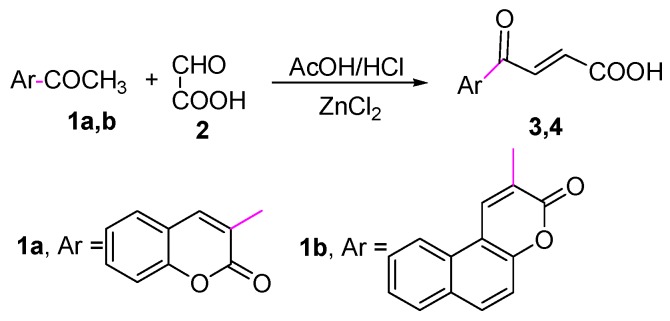
Synthesis of compounds **3** and **4**.

In the present investigation we report the syntheses of some new coumarin derivatives having antitumor and antioxidant activities. 4-Oxo-4-(2-oxo-2*H*-chromen-3-yl)but-2-enoic acid (**3**) was synthesized by Tolstoluzhsky *et al.* [38] using glyoxalic acid and acetic anhydride in the presence of ytterbium triflate as a catalyst under microwave conditions. Herein we synthesized the target compounds **3** and **4** by the reaction of 3-acetyl-2*H*-chromen-2-one (**1a**) and 2-acetyl-3*H*-benzo[*f*]-chromen-3-one (**1b**) with glyoxalic acid (**2**) in acetic acid/HCl to afford 4-oxo-4-(2-oxo-2*H*-chromen-3-yl)but-2-enoic acid (**3**) and 4-oxo-4-(3-oxo-3*H*-benzo[*f*]chromen-2-yl)but-2-enoic acid (**4**), respectively (Scheme 1). The IR spectra of compounds **3** and **4** showed characteristic absorption bands at 3446, 3476 cm^−1^ attributable to OH and also in the range 1730–1656 and 1742–1642 cm^−1^ attributable to C=O, respectively. ^1^H-NMR spectra of compounds **3** and **4** showed exchangeable signals at δ 12.51, 9.40 ppm, respectively, assigned to the OH protons. The mass spectra of compounds **3** and **4** showed the molecular ion peak at *m*/*z* 244 and a [M + 2]^+^ peak at *m/z* 296, respectively which coincide with the molecular weights supporting the proposed identity of the structures.

**Figure 2:**
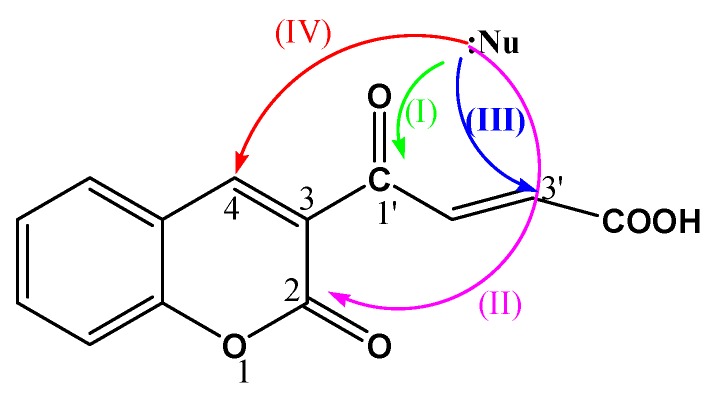
Possible sites of nucleophile attack on coumarin derivatives.

In principle, a nucleophile might be expected to attack a coumarin substrate at any of the electrophilic centers, C-1′ (I) C-2 (II), C-3′ (III) or C-4 (IV) as illustrated in Figure 2.

**Scheme 2:**
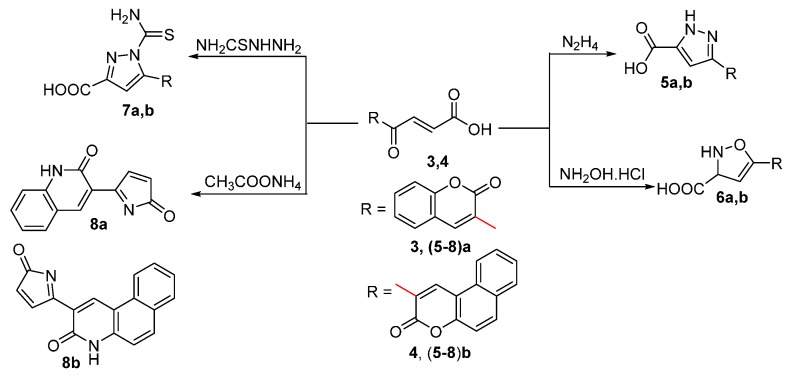
Synthesis of compounds (**5**–**8**)**a,b**.

The reactions of compounds **3** and **4** with various nitrogen nucleophiles have been shown to proceed with a high degree of regioselective at the exocyclic acrylic center at C-3′ followed by *exo-trig* ring closure to afford the substituted coumarin products (Scheme 2). Aroylacrylic acid derivatives can be used in a wide range of the addition reaction. The strong electron-attracting power of the aryl carbonyl group enhances the reactivity of the adjacent double bond-function and promotes the nucleophilic addition at this center. Simultaneous or subsequent cyclization of adducts gives access to various 5- or 6-membered cyclic structures. All the structures were proved by spectroscopic studies.

Compounds **3** and **4** were reacted with hydrazine hydrate [39] to yield the pyrazolecarboxylic acid derivatives **5a** and **5b**, respectively, via aza Michael addition followed by cyclization (Scheme 2). The spectroscopic data are consistent with the proposed structures. The IR spectra of compounds **5a** and **5b** showed bands for acidic OH and NH protons at 3440, 3217, 3400 and 3283 cm^−1^ respectively. The ^1^H-NMR spectra showed the appearance of OH protons at δ 11.03 and 12.82 ppm and NH protons at 7.01 and 6.99 ppm for compounds **5a** and **5b** respectively indicated the presence of the carboxylic and the NH groups.

Similarly, treatment of compounds **3** and **4** with hydroxylamine hydrochloride in pyridine [39] afforded compounds **6a** and **6b**. The structures of compounds **6a** and **6b** were deduced from their elemental analysis and spectral data. The IR spectra showed bands for OH, NH and C=O at 3393, 3380, 3204, 3128, 1720, 1728, 1710 and 1718 cm^−1^ respectively. The ^1^H-NMR spectra showed the presence of exchangeable OH and NH protons at δ 11.90, 11.52, 10.10 and 6.93 ppm respectively, beside signals for aromatic and =CH protons (*cf.* Experimental Section).

Compounds **3** and **4** were also reacted with thiosemicarbazide [40] to yield compounds **7a** and **7b**. The structures of compounds **7a** and **7b** were deduced by spectroscopic data. The IR spectra showed bands for OH, NH and C=O at 3455, 3382, 3397, 1711, 1712, 1647 and 1653 cm^−1^, respectively. The ^1^H-NMR spectra showed the presence of exchangeable OH, NH_2_ at δ 12.26, 12.0, 5.71 and 6.48 ppm, respectively, beside signals for aromatic and =CH protons (*cf.* Experimental Section).

Compounds **8a** and **8b** were formed when a mixture of compounds **3** and/or **4** was fused with ammonium acetate for 3h in the absence of solvent [41]. Proof of the structures of compounds **8a** and **8b** were based on spectral data. The IR spectra showed bands attributed to NH, and C=O in the range 3181–3385 and 1642–1713 respectively. Also the ^1^H-NMR showed signals at δ 9.57 and 9.71 ppm for NH protons beside signals in the range of 7.95–8.16, 6.97–8.06 ppm attributed to coumarin, aromatic and HC=CH protons, respectively. The ammonium acetate was reacted with the carboxylic acid group followed by ring closure, and also with the coumarin ring to give the oxopyrrolylquinolinone and the oxopyrrolylbenzoquinolinone derivatives **8a** and **8b**, respectively.

**Scheme 3:**
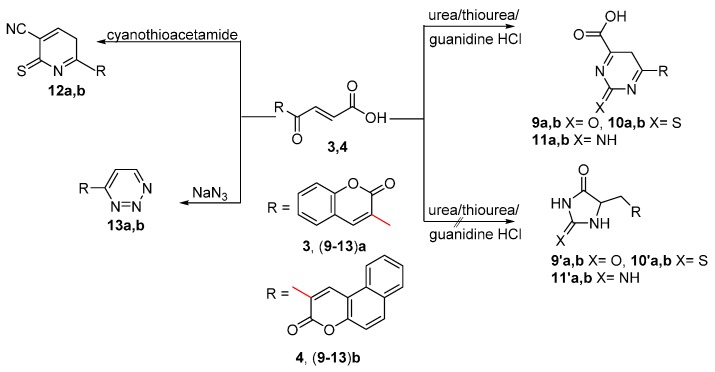
Synthesis of compounds (**9**–**13**)**a**,**b**.

Furthermore, the reaction of compounds **3** and **4** with urea, thiourea [40] and guanidine hydrochloride in DMF afforded products whose spectra (IR, ^1^H-NMR and MS) and elemental analyses data are consistent with compounds (**9**–**11**)**a**,**b** rather than compounds (**9′**–**11′**)**a**,**b** (Scheme 3). The reaction took place via addition of the nitrogen nucleophile on the α-carbon atom followed by *exo-trig* ring closure on the ketonic carbonyl carbon atom and dehydrogenation to give compounds (**9**–**11**)**a**,**b**. The structures of compounds (**9**–**11**)**a**,**b** were confirmed by spectroscopic data. The other products (**9′**–**11′**)**a**,**b** were discounted on the basis of the spectral data. The IR spectra of compounds (**9**–**11**)**a**,**b** showed bands for OH and C=O in the range 3403–3455 and 1642–1653 cm^−1^ respectively. The ^1^H-NMR spectra showed the appearance of CH_2_ and OH protons in the range 1.20–1.23 and 9.78–11.23 ppm respectively for compounds (**9**–**11**)**a**,**b** which indicated the presence of the methylene and the carboxylic groups. The mass spectrum of compound **9a** showed the molecular ion peak at *m*/*z* 284 (14.42%) which is in a good agreement with the molecular formula C_14_H_8_N_2_O_5_. Compound **10a** failed to react with benzaldehyde in the presence of a mixture of acetic acid, acetic anhydride and zinc chloride, which is a good evidence for the existence of compound **10a** and not **10′a**.

Cyanothioacetamide reacted with compounds **3** and **4** to give compounds **12a,b**. Cyanothioacetamide [42] has three nucleophile sites: the methylene carbon, the amino group and the sulfur atom. On the whole, under cyclocondensation or cycloaddition conditions, cyanothio-acetamide acts as a C,N-, C,S- or S,N- binucleophile. In this case the reaction occurred via Michael addition reaction followed by ring closure, elimination of water and hydrogen molecules to give compounds **12a** and **12b**. The IR spectra showed bands attributable to C≡N and C=O groups at 2209, 2207, 1713 and 1705 cm^−1^ respectively, with the absence of bands attributable to OH and NH groups. The ^1^H-NMR spectra showed the presence of signals for aromatic, =CH, coumarinic hydrogen, and CH_2_ protons at 6.22–7.94, 6.81–7.95, 1.72 and 1.20 ppm, respectively. The mass spectra of compounds **12a** and **12b** showed the molecular ion peak at *m*/*z* 280 and a [M + 2]^+^ ion at *m/z* 332, respectively, which agree well with the suggested structures.

On the other hand, sodium azide reacted with compounds **3** and **4** to yield the triazine derivatives **13a** and **13b**. The reaction occurred via addition reaction on the carbon double bond followed by ring closure, elimination of water molecule and decarboxylation to give the stable compounds **13a** and **13b**. The structures of compounds **13a** and **13b** were confirmed based on spectral data. The IR showed bands for C=O and C=N at 1718, 1707 and 1631, 1624 cm^−1^, respectively and the absence of bands corresponding to OH and NH groups. The ^1^H-NMR spectra showed the absence of signals corresponding to NH and OH protons, and showed signals for coumarin, aromatic and two =CH protons in the range 8.89–6.42 ppm.

**Scheme 4:**
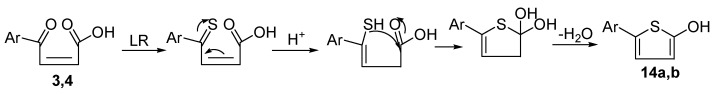
Mechanistic route of compounds **14a,b**.

Lawesson’s reagent is a powerful, mild, and versatile thiation agent that efficiently converts oxygen functionalities into their thio*-*analogs. This reagent has been used for the cyclization of compounds containing at least two oxygen functionalities [43] Lawesson’s reagent acts as a sulfurizing agent as well as dehydrating agent. Compounds **3** and **4** were refluxed with Lawesson’s reagent in acetonitrile to give compounds **14a** and **14b** (Scheme 4).

The reaction seems to produce like Paal-Knorr synthesis mechanism to produce the thiophene ring (Scheme 4). Proof of the structures of compounds **14a** and **14b** were based on spectral data. The IR spectra showed bands attributed to OH and C=O at 3557, 3428 and 1724 cm^−1^, respectively. The ^1^H-NMR showed signals for aromatic, coumarin proton, two =CH and OH protons at 7.61–7.54, 7.37–8.64, 6.91, 6.96, 7.14 and 6.20 ppm respectively. The mass spectra of compounds **14a** and **14b** showed the molecular ion peaks at *m*/*z* 261 and 310, respectively, which coincide with their molecular weights.

**Scheme 5:**
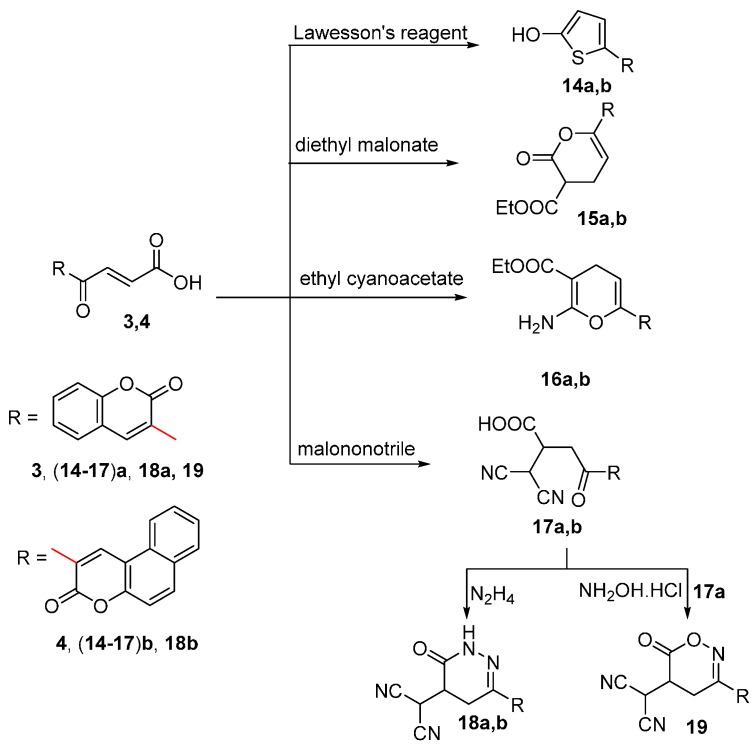
Synthesis of compounds **14**–**19**.

The present study was extended to investigate the chemical behavior of compounds **3** and **4** towards some acyclic carbon nucleophiles containing an active methylene group between two carbonyl groups (Scheme 5). There have been reports on Michael reactions catalyzed by K_2_CO_3_ under phase transfer catalysis [44]. To some extent these mild conditions can minimize the reversibility of the Michael addition reaction [45] and other side reactions, thus improved yields can be achieved. Therefore, boiling [39] compounds **3** and **4** with diethyl malonate and/or ethyl cyanoacetate under PTC reaction conditions in the presence of K_2_CO_3_ and tetrabutyl-*n*-ammonium bromide afforded compounds (**15**,**16**)**a**,**b**, respectively. The pyranone derivatives (**15**,**16**)**a**,**b** were formed via Michael addition reaction of the carbanion at the exo-double bond followed by cyclization and decarboxylation. The structures of compounds (**15**,**16**)**a**,**b** were elucidated by spectroscopic data. The IR of compounds **15a** and **15b** showed bands at 1715, 1716 cm^−1^ for ester groups, respectively. The ^1^H-NMR of compounds **15a** and **15b** showed triplet signals attributed to CH_3_ at 0.93 ppm and quartet signal for OCH_2_ at 3.16–3.18 and 3.12–3.18 ppm, respectively beside signals for CH_2_, CH, =CH and aromatic protons in the range 1.22–1.35, 1.50–1.62, 6.50–8.16 ppm, respectively. Also the spectra showed a signal for the coumarin proton at 8.59 and 8.53 ppm, respectively. The IR of compounds **16a** and **16b** showed bands at 3418, 3228, 3409 and 3242 cm^−1^ attributed to NH_2_ and also bands at 1712 cm^−1^ for C=O group. The ^1^H-NMR of compounds **16a** and **16b** showed signals for CH_3,_ CH_2_, OCH_2_, NH_2_, =CH, aromatic and coumarin protons.

Meanwhile the reaction of compounds **3** and **4** with malononitrile [46] under the same conditions afforded the open structures **17a** and **17b**, respectively. The structures of compounds **17a** and **17b** were elucidated by spectroscopic data. The IR spectra showed bands for OH, C≡N and C=O at 3434, 2209, 2210, 1646 (broad) and 1632 (broad) cm^−1^, respectively. Also the ^1^H-NMR spectra showed signals for OH protons at 12.22 and 12.56 ppm, respectively, in addition to signals attributed to CH, CH_2_, aromatic and coumarin protons. The structures of compounds **17a** and **17b** were deduced chemically by their reaction with hydrazine hydrate [46] and also the reaction of **17a** with hydroxylamine hydrochloride to give compounds **18a**, **18b** and **19**, respectively. The reaction was proceeded via condensation reaction of the NH_2_ group with the ketonic group followed by 6-*exo-trig* cyclization. The structures of compounds **18a**, **18b** and **19** were proved by spectroscopic tools. The IR spectra showed bands in the range 2207–2211, 1627–1713 cm^−1^ attributable to C≡N and C=O groups, respectively, in addition to bands at 3362 and 3399 cm^−1^ attributable to NH for compounds **18a** and **18b**, respectively. The ^1^H-NMR spectra of compounds **18a** and **18b** showed signals in the range 1.20–1.27, 1.90–1.97 and 2.21–2.75 ppm attributed to CH, CH_2_ and CH(CN)_2_, respectively, in addition to signals attributed to aromatic, coumarin and NH protons. The ^1^H-NMR spectrum of compound **19** showed signals at 1.22–1.25, 2.26–2.30, 2.72, 7.05–8.34 and 8.77 ppm attributed to CH_2_, 2CH, aromatic and coumarin protons, respectively.

### 2.2. Pharmacological Activity

#### 2.2.1. Cytotoxic Activity Using an *In Vitro* Ehrlich Ascites Assay

Out of the newly synthesized compounds, twenty three analogs were selected to be evaluated for their *in vitro* cytotoxic effect against a panel of four human tumor cell lines namely: hepatocellular carcinoma (liver) HepG2, colon cancer HCT-116, human prostate cancer PC3, and mammary gland breast MCF-7 cancer cell lines (Table 1). In general, the activity observed by all of these molecules ranged from very strongly to weakly cytotoxic. The results revealed that 12 of the tested compounds (**5a**,**b**, **7a**,**b**, **9a**,**b**, **11b**, **13b**, **15a**,**b**, **16a** and **18a**) exhibited varying degrees of inhibitory activity towards the four tested tumor cell lines, ranging from strong to very strong. As for the activity against hepatocellular carcinoma HePG2, the highest cytotoxic activity was displayed by compounds **7a**,**b** and **15b** which showed IC_50_ values of 10.8 ± 0.88, 9.3 ± 0.58 and 8.2 ± 0.45 μg/mL, respectively. Remarkably strong inhibitory activity ranging from 11.0–19.7 was also seen for compounds **5a**, **9a**,**b**, **13b** **15a** and **18a**, while **3**, **5b**, **10a**, **11b**, **16a**,**b** and **19** also showed moderate activity and **4**, **10b**, **11a**, **13a**, **17a**,**b** and **18b** had weak activity.

As for the activity against colon cancer HCT-116 cell line, the highest cytotoxic activities were displayed by compounds **7a**,**b**, **9a** and **15b** which showed IC_50_ at 10.4 ± 0.94, 4.8 ± 0.18, 9.4 ± 0.97 and 9.7 ± 0.84 μg/mL, respectively. Compound **7b** showed higher activity than the reference (5-Fu 5.3 ± 0.14). Remarkable strong inhibitory activities were also demonstrated by compounds **5a**, **9b**, **11b**, **13b**, **15a**, **16a** and **18a** ranging 10.4–20.0 μg/mL. Also compounds **3**, **4**, **5b**, **10a**, **16b**, **17b** and **19** showed moderate activity, while compounds **10b**, **11a**, **13a**, **17a** and **18b** showed weak activity.

Compounds **7a**, **13b** and **15b** were found to be the most potent derivatives overall the tested compounds against human prostate cancer cell line PC3 with IC_50_ 9.6 ± 0.82, 10.8 ± 0.79 and 8.7 ± 0.45 μg/mL, respectively. Compound **15b** is almost equipotent as 5-fluorouracil (IC_50_ = 8.3 ± 0.25 μg/mL). Also compounds **5a**, **7b**, **9a**,**b**, **15a**, **16a** and **18a** were strongly active with IC_50_ = 16.2 ± 1.56, 11.1 ± 1.13, 14.5 ± 1.30, 18.0 ± 1.96 13.7 ± 0.94, 13.4 ± 0.96 and 16.1 ± 1.08 μg/mL, respectively. On the other hand compounds **3**, **4**, **5b**, **11b**, **16b** and **19** showed moderate activity, while compounds **10a**,**b**, **11a**, **13a**, **17a,b** and **18b** showed weak activity.

Whilst compounds **7a,b** and **11b** showed very strong activity towards mammary gland (breast) MCF-7 with IC_50_ 10.6 ± 0.92, 7.8 ± 0.67 and 5.6 ± 0.43 μg/mL, respectively, compounds **5a**,**b**, **9a**,**b** **15a**,**b**, **16a** and **18a** displayed strong activity, with IC_50_ = 15.7 ± 1.24, 15.8 ± 1.08, 12.0 ± 1.14, 20.4 ± 1.56, 17.6 ± 1.37, 14.1 ± 1.21, 19.9 ± 2.14 and 13.2 ± 0.76 μg/mL, respectively. On the other hand compounds **3**, **4**, **10a**, **13b**, **16b**, **17b** and **19** showed moderate activity, while compounds **10b**, **11a**, **13a**, **17a** and **18b** showed weak activity. Compound **11b** is almost equipotent to 5-fluorouracil (IC_50_ = 5.4 ± 0.21 μg/mL). Compounds containing the coumarin ring showed higher cytotoxic activity than their analogs containing the benzocoumarin ring (the relative viabilities of cells (%) for the reference and the tested compounds are listed in Tables S1 and S2 and Figures S1–S24 as Supplementary Materials).

#### 2.2.2. Antioxidant Activity Using ABTS Inhibition

Twenty three compounds were tested for antioxidant activity reflected as the ability to inhibit oxidation in rat brain and kidney homogenates (Table 2).

Compounds **5b**, **7b**, **9b**, **11b** and **13b** showed very high inhibitions of 84.4, 84.6, 84.6, 84.4 and 84.6%, respectively. Compounds **4,** **5a**, 7**a**, **9a**, **15b**, **17b** and **18a** showed high inhibitions of 72.9, 69.9, 75.3, 68.9, 75.5, 69.3 and 66.0%, respectively. In addition the rest of the compounds **3**, **10a**,**b**, **11a**, **13a**, **15a**, **16a**,**b**, **17a**, **18b** and **19** exhibited moderate to weak antioxidant activity ranging from 59.5–39.8%. Compounds containing the benzocoumarin ring showed higher antioxidant activity than their analogs containing the coumarin ring.

#### 2.2.3. Bleomycin-Dependent DNA Damage

Bleomycins are a family of glycopeptide antibiotics routinely used as antitumor agents. The bleomycin assay has been adopted for assessing the pro-oxidant effect of food antioxidants. The antitumor antibiotic bleomycin binds iron ions and DNA. The bleomycin-iron complex degrades DNA when heated with thiobarbituric acid (TBA) to yield a pink chromogen. Upon the addition of suitable reducing agents the antioxidant competes with DNA and diminishes chromogen formation [47].

To show the mechanism of action of the twenty three tested compounds, their protective activity against DNA damage induced by the bleomycin-iron complex was examined. The results (Table 2) showed that compounds **11b** (0.072) and **15b** (0.076) were equipotent to ascorbic acid (0.073). Consequently, they have the ability to protect DNA from the damage induced by bleomycin. Compounds **3**, **5b**, **7a**,**b**, **9a**,**b**, **15a**, **16a** and **18a** meanwhile showed high protection against DNA damage induced by the bleomycin-iron complex ranged from 0.081–0.099. On the other hand, the rest of the compounds exhibited weak activities. Thus, all the tested compounds diminish the chromogen formation between the damage DNA and TBA with different activity. Compounds containing the benzocoumarin ring showed higher activity against DNA damage than their analogs containing the coumarin rings.

#### 2.2.4. Structure Activity Relationships

The antitumor activity of natural and synthetic coumarin derivatives has been extensively explored by many researchers [30,48,49,50] and it has been proven that coumarins, depending on their structure, can act on various tumor cells by different mechanisms, inhibiting the enzyme telomerase, protein kinase activity and downregulating oncogene expression or inducing caspase-9-mediated apoptosis, suppressing cancer cell proliferation by arresting the cell cycle in G0/G1 phase, G2/M phase and affecting the p-groups of cancer cells [51,52].

By comparing the experimental cytotoxicity of the compounds reported in this study to their structures, the following structure activity relationships (SAR) are postulated:

- The cytotoxic activity of compounds **3**, **4** is due to the presence of the coumarin moiety and also the formation of intermolecular hydrogen bonds between the OH [53] and DNA bases.
- The cytotoxic activity of compounds **7a**,**b** is due to the presence of the coumarin moiety and also the formation of intermolecular hydrogen bonds of OH and NH_2_ groups with DNA bases [52]. Introducing the pyrazole carbothioamide moiety [25] also enhances the cytotoxic activity of compounds **7a**,**b**.
- The cytotoxic activity of compounds **5a**,**b** is due to the presence of the coumarin and the pyrazole carboxylic acid moieties [42] and also the formation of intermolecular hydrogen bonds between the OH and DNA bases. Compounds **7a**,**b** demonstrated better activity compared to compounds **5a**,**b**, probably due to the presence of the carbothioamide (S=CNH_2_) moiety.
- Compounds **9a**,**b** showed strong activity due to the presence of the coumarin and the pyridazinone rings.
- Compounds **15a**,**b** and **16a** showed strong and very strong activity due to the presence of the coumarin and the pyran rings.
- Compounds **18a** showed strong activity due to the presence of the coumarin and the pyridazine rings, and also the presence of two cyano groups.

## 3. Experimental Section

### 3.1. General Information

All melting points were measured on a Gallenkamp melting point apparatus and are uncorrected. The infrared spectra were recorded using potassium bromide disks on a Mattson FTIR infrared spectrophotometer (Mattson, New York, NY, USA). ^1^H-NMR spectra were run at 300 MHz, on a Varian Mercury VX-300 NMR spectrometer (Bruker, Rheinstetten, Germany), using TMS as an internal standard in deuterated dimethylsulphoxide. Chemical shifts δ are quoted in ppm and *J* in Hz. The mass spectra were recorded on a GCMS-QP-1000EX mass spectrometer (Shimadzu, Kyoto, Japan) at 70 e.V. All the spectral measurements were carried out at the Microanalytical Center of Cairo University, Cairo, Egypt and the Main Defense Chemical Laboratory, Cairo, Egypt. The elemental analyses were carried out at the Microanalytical center of Ain Shams University, Cairo, Egypt. The pharmaceutical activity assays were carried out at Pharmacology Department, Faculty of Pharmacy, EL-Mansoura University, EL-Mansoura, Egypt. All the chemical reactions were monitored by TLC.

### 3.2. Synthesis

#### 3.2.1. General Procedure for the Synthesis of Compounds **3** and **4**

A mixture of compound **1** and/or **2** (0.012 mol) and glyoxalic acid (0.81 g, 0.011 mol) in glacial acetic acid (20 mL) containing HCl (1–2 mL) in the presence of ZnCl_2_ (0.5 g) was heated under reflux for 8 h. The reaction mixture was cooled and then poured on hot water to dissolve the ZnCl_2_. The separated solid was filtered off and washed with water, dried and recrystallized from the proper solvent to give compounds **3** and **4**, respectively.

*4-Oxo-4-(2-oxo-2H-chromen-3-yl)but-2-enoic Acid* (**3**). Pale brown crystals; yield (1.8 g, 74%); m.p. 200–203 °C; methanol. IR (KBr) cm^−1^: 3446 (OH), 1730–1656 (C=O), 1609 (C=C). ^1^H-NMR (DMSO-*d*_6_) δ: 7.19–8.66 (m, 6H, 4ArH, 2H olefinic), 9.58 (s, 1H, coumarin), 12.51 (s, 1H, OH D_2_O exchangeable). MS *m/z*: 244 ([M]^+^) (42.9), 199 (42.9), 145 (78.6), 132 (21.4), 131 (71.4), 92 (57.1), 78 (14.3). Anal. Calcd. for C_13_H_8_O_5_: C, 63.94; H, 3.30. Found: C, 63.81; H, 3.32.

*4-Oxo-4-(3-oxo-3H-benzo[f]chromen-2-yl)but-2-enoic Acid* (**4**). Brown crystals; yield (2.12 g, 72.2%); m.p. 229–231 °C; dioxane/H_2_O (3:1). ^1^H-NMR (DMSO-*d*_6_) δ: 7.60–8.11 (m, 8H, 6 ArH, 2H olefinic), 8.34 (s, 1H, coumarin), 9.40 (s, 1H, OH D_2_O exchangeable). IR (KBr) cm^−1^: 3476 (OH), 1742–1642 (C=O), 1627 (C=C). MS *m/z*: 296 ([M + 2]^+^) (4.78), 249 (7.19), 224 (10.25), 223 (76.82), 198 (14.10), 196 (100.0), 181 (24.30), 168 (63.44), 165 (23.65), 156 (35.56), 153 (17.16), 144 (66.47), 127 (41.76), 72 (23.55), 62 (23.57), 56 (20.29), 46 (25.09). Anal. Calcd. for C_17_H_10_O_5_: C, 69.39; H, 3.43. Found: C, 69.45; H, 3.46.

#### 3.2.2. General Procedure for the Synthesis of Compounds **5a** and **5b**

A mixture of compound **3** and/or **4** (0.01 mol), hydrazine hydrate, (0.50 mL, 0.01 mol) in DMF (20 mL) was refluxed for 3 h, cool, poured on water, The solid that separated was filtered off, dried and recrystallized from the proper solvent to give compounds **5a** and **5b** respectively.

*3-(2-Oxo-2H-chromen-3-yl)-1H-pyrazole-5-carboxylic Acid* (**5a**). Brown crystals; yield (2.24 g, 87.5%); m.p. > 300 °C; DMF/H_2_O (3:1). ^1^H-NMR (DMSO-*d*_6_) δ: 7.01 (br.s, 1H, NH D_2_O exchangeable,), 6.97–7.96 (m, 5H, 4ArH, =CH), 9.01 (s, 1H, coumarin), 11.03 (br.s, 1H, OH D_2_O exchangeable). IR (KBr) cm^−1^: 3440 (OH), 3217 (NH), 1720 (C=O coumarin), 1674 (C=O acid), 1625 (C=N). MS *m/z*: 256 ([M]^+^) (0.00), 240 (10.52), 149 (10.71), 147 (6.00), 136 (39.95), 122 (8.53), 120 (28.24), 93 (66.40), 91 (100), 74 (8.10), 72 (22.02), 60 (40.16), 58 (12.49), 56 (44.58), 54 (50.41), 46 (20.89). Anal. Calcd. for C_13_H_8_N_2_O_4_: C, 60.94; H, 3.15; N, 10.93. Found: C, 60.79; H, 18; N, 10.95.

*3-(3-Oxo-3H-benzo[f]chromen-2-yl)-1H-pyrazole-5-carboxylic Acid* (**5b**). Brown crystals; yield (2.47 g, 81%); m.p. > 300 °C; methanol. ^1^H-NMR (DMSO-*d*_6_) δ: 6.99 (br.s, 1H, NH, D_2_O exchangeable,), 7.11–8.10 (m, 7H, 6ArH, =CH), 8.86 (s, 1H, coumarin), 12.82 (s, 1H, OH, D_2_O exchangeable). IR (KBr) cm^−1^: 3400 (OH), 3283 (NH), 1722 (C=O coumarin), 1671 (C=O acid), 1625 (C=N). MS *m/z* (%): 306 ([M]^+^) (0.00), 262 (0.22), 169 (25.84), 170 (11.60), 144 (28.16), 143 (61.74), 128 (18.61), 127 (11.35), 116 (21.79), 115 (100), 114 (20.20), 68 (0.60). Anal. Calcd. for : C_17_H_10_N_2_O_4_: C_,_ 66.67; H, 3.29; N, 9.15. Found: C, 66.69; H, 3.30; N, 9.16.

#### 3.2.3. S General Procedure for the Synthesis of Compounds **6a** and **6b**

A mixture of compound **3** and/or **4** (0.01 mol), and hydroxylamine hydrochloride (0.69 g, 0.01 mol) in pyridine (20 mL) was refluxed for 3 h, cool, poured on ice/HCl, The solid that separated was filtered off, dried and recrystallized from the proper solvent to give compounds **6a** and **6b** respectively.

*5-(2-Oxo-2H-chromen-3-yl)-2,3-dihydroisoxazole-3-carboxylic Acid* (**6a**). Reddish brown crystals; yield (1.49 g, 57.9%); m.p. 230–232°C; methanol/H_2_O (2:1). ^1^H-NMR (DMSO-*d*_6_) δ: 4.05 (d, 1H, CH), 6.91–7.80 (m, 5H, 4ArH, =CH), 9.05 (s, 1H, coumarin), 10.10 (s, 1H, NH D_2_O exchangeable), 11.90 (s, 1H, OH, D_2_O exchangeable). IR (KBr) cm^−1^: 3393 (OH), 3204 (NH), 1720 (C=O coumarin), 1710 (C=O acid), 1610 (C=N). MS *m/z*: 260 ([M + 1]^+^) (4.98), 243 (15.22), 215 (25.33), 146 (39.80), 118 (32.05), 115 (29.27), 104 (19.70), 91 (52.46), 78 (69.66), 71 (47.66), 57 (68.36), 55 (57.48), 46 (43.66), 45 (100). Anal. Calcd. for C_13_H_9_NO_5_: C, 60.24; H, 3.50; N, 5.40. Found: C, 61.12; H, 3.49; N, 5.37.

*5-(3-Oxo-3H-benzo[f]chromen-2-yl)-2,3-dihydroisoxazole-3-carboxylic Acid* (**6b**). Pale brown crystals; yield (1.79 g, 58.25%); m.p. 174–175 °C; methanol. ^1^H-NMR (DMSO-*d*_6_) δ*:* 4.13 (d, 1H, CH), 6.93 (s, 1H, NH D_2_O exchangeable), 7.18–8.79 (m, 7H, 6ArH, =CH), 9.03 (s, 1H, coumarin), 11.52 (s, 1H, OH D_2_O exchangeable). IR (KBr) cm^−1^: 3380 (OH), 3128 (NH), 1728 (C=O coumarin), 1718 (C=O acid), 1627 (C=N). MS *m/z*: 309 ([M]^+^) (0.56), 170 (13.27), 144 (11.23), 141 (18.27), 140 (17.02), 115 (22.51), 114 (33.80), 79 (100), 78 (17.90), 71 (16.18), 70 (15.40), 57 (30.65). Anal. Calcd. for C_17_H_11_NO_5_: C, 66.02; H, 3.58; N, 4.53. Found: C, 66.11; H, 3.60; N, 4.52.

#### 3.2.4. General Procedure for the Synthesis of Compounds **7a** and **7b**

A mixture compound **3** and/or **4** (0.01 mol), thiosemicarbazide hydrochloride, (1.27 g, mL, 0.01 mol) in DMF (20 mL) was refluxed for 3 h, cool, poured on water, The solid that separated was filtered off, dried and recrystallized from the proper solvent to give compounds **7a** and **7b** respectively.

*1-Carbamothioyl-5-(2-oxo-2H-chromen-3-yl)-1H-pyrazole-3-carboxylic Acid* (**7a**). Brown crystals; yield (2.32 g, 73.8%); m.p. 253–255 °C; DMF/H_2_O (3:1). ^1^H-NMR (DMSO-*d*_6_) δ: 55.71 (s, 2H, NH_2_, D_2_O exchangeable), 6.93–7.52 (m, 5H, ArH, =CH), 7.94 (s, 1H, coumarin), 12.26 (s, 1H, OH, D_2_O exchangeable). IR (KBr) ν_max_/cm^−1^: 3455 (OH), 3406, 3382 (NH_2_), 1711 (C=O coumarin), 1647 (C=O acid), 1607 (C=N). MS *m/z*: 315 ([M]^+^) (0.00), 314 ([M − 1]^+^) (3.07) 299 (2.35), 145 (3.65), 117 (3.85), 74 (6.31), 73 (100), 60 (25.43), 58 (15.94), 57 (82.07). Anal. Calcd for C_14_H_9_N_3_O_4_S: C, 53.33; H, 2.88; N, 13.33; S, 10.17. Found: C, 53.70; H, 2.78; N, 13.40; S, 10.05.

*1-Carbamothioyl-5-(3-oxo-3H-benzo[f]chromen-2-yl)-1H-pyrazole-3-carboxylic Acid* (**7b**). Brown crystals; yield (2.38 g, 65.4%); m.p. 220–223 °C; DMF/H_2_O (3:1). ^1^H-NMR (DMSO-*d*_6_) δ: 6.48 (s, 2H, NH_2_, D_2_O exchangeable), 7.26–8.62 (m, 7H, 6ArH, =CH) 8.65 (s,1H coumarin), 12.0 (s, 1H, OH, D_2_O exchangeable). IR (KBr) cm^−1^: 3455 (OH), 3419, 3397 (NH_2_), 1712 (C=O coumarin), 1653 (C=O acid), 1625 (C=N). MS *m/z*: 367 ([M + 2]^+^) (0.93), 340 (12.65), 115 (18.67), 73 (100), 58 (9.22). Anal*.* Calcd for C_18_H_11_N_3_O_4_S: C, 59.17; H, 3.03; N, 11.50; S, 8.78. Found: C, 59.28; H, 3.05; N, 11.53; S, 8.80.

#### 3.2.5. General Procedure for the Synthesis of Compounds **8a** and **8b**

A mixture of compound **3** and/or **4** (0.01 mol) and ammonium acetate (1.54 g, 0.02 mol) was fused at 150–170 °C for 4 h. The reaction mixture poured onto ice/HCl. The solid that separated was filtered off, dried and recrystallized from the proper solvent to give compounds **8a** and **8b** respectively.

*3-(5-Oxo-5H-pyrrol-2-yl)quinolin-2(1H)-one* (**8a**). Brown crystals; yield (1.69 g, 75.4%); m.p. > 300 °C; DMF/H_2_O (3:1). ^1^H-NMR (DMSO-*d*_6_) δ: 4.91 (d, 1H, *CH*=CHCO), 5.12 (d, 1H, CH=*CH*CO), 7.00–7.95 (m, 5H, 4ArH,1H, coumarin); 9.57 (s, 1H, NH, D_2_O exchangeable). IR (KBr) cm^−1^:3181 (NH), 1710, 1660 (C=O), 1607 (C=N). MS *m/z*: 224 ([M]^+^) (0.00), 225 ([M + 1]^+^) (21.10), 145 (20.13), 104 (12.53), 81 (24.42), 73 (100), 57 (41.93). *Anal.* Calcd for C_13_H_8_N_2_O_2_: C, 69.64; H, 3.60; N, 12.49. Found: C, 69.80; H, 3.78; N, 12.46.

2-(5-Oxo-5*H*-pyrrol-2-yl)benzo[*f*]quinolin-3(4*H*)-one (**8b**). Brown crystals; yield (1.89 g, 69%); m.p. 254–256 °C; DMF/EtOH (2:1). ^1^H-NMR (DMSO-*d*_6_) δ: 7.06–8.16 (m, 8H, 6ArH, 1H, coumarin, 2H, 2=CH), 9.71 (s, 1H, NH, D_2_O exchangeable). IR (KBr) cm^−1^:1713, 1642 (C=O), 1626 (C=N). MS *m/z*: 274 ([M]^+^) (0.00), 196 (46.29), 195 (13.87), 170 (16.14), 156 (22.43), 128 (33.78), 80 (100), 74 (6.75). Anal. Calcd. for C_17_H_10_N_2_O_2_: C, 74.44; H, 3.67; N, 10.21. Found: C, 74.63; H, 3.64; N, 10.31.

#### 3.2.6. General Procedure for the Synthesis of Compounds **(9**–**12)a,b**

A mixture of compound **3** and/or **4** (0.01 mol), urea, thiourea, guanidine hydrochloride and cyano thioactamide (0.01 mol) in DMF (20 mL) was refluxed for 3 h, cool, poured on water, The solid that separated was filtered off, dried and recrystallized from the proper solvent to give compounds **9**–**12**(**a,b**), respectively.

*2-Oxo-6-(2-oxo-2H-chromen-3-yl)-2,5-dihydropyrimidine-4-carboxylic Acid* (**9a**). Brown crystals; yield (2.08 g, 73.4%); m.p. 237–239 °C; dioxane/H_2_O (3:1). ^1^H-NMR (DMSO-*d*_6_) δ: 1.22 (s, 2H, CH_2_ methylene), 7.03–8.59 (m, 4H, ArH), 9.01 (s, 1H, coumarin), 11.23 (s, 1H, OH exchangeable with D_2_O). IR (KBr) cm^−1^: 3455 (OH), 1720–1674 (C=O), 1624 (C=N), 1610 (C=C). MS *m/z*: 284 ([M]^+^, 14.42), 240 (15.34), 212 (23.51), 190 (14.49), 184 (16.77), 170 (14.97), 98 (37.20), 60 (100). Anal. Calcd. for C_14_H_8_N_2_O_5_: C, 59.16; H, 2.84; N, 9.86. Found: C, 59.20; H, 2.90; N, 9.75.

*2-Oxo-6-(3-oxo-3H-benzo[f]chromen-2-yl)-2,5-dihydropyrimidine-4-carboxylic Acid* (**9b**). Brown crystals; yield (2.18 g, 65.47%); m.p. 274–276 °C; DMF/H_2_O (3:1). ^1^H-NMR (DMSO-*d*_6_) δ: 1.20 (s, 2H, CH_2_ methylene), 6.61–8.93 (m, 6H, ArH), 9.05 (s, 1H, coumarin), 10.25 (s, 1H, OH, D_2_O exchangeable). IR (KBr) cm^−1^: 3437 (OH), 1720–1642 (C=O), 1626 (C=N). MS *m/z*: 334 ([M]^+^, 0.00), 289 (47.5), 196 (27.51), 170 (10.0), 158 (42.5), 96 (44.3), 80 (100). Anal. Calcd. for C_18_H_10_N_2_O_5_: C, 64.67; H, 3.02; N, 8.38. Found: C, 64.82; H, 3.04; N, 8.35.

*6-(2-Oxo-2H-chromen-3-yl)-2-thioxo-2,5-dihydropyrimidine-4-carboxylic Acid* (**10a**). Brown crystals; m.p. 150–153 °C; yield (2.38 g, 79.4%); dioxane/H_2_O (3:1). ^1^H-NMR (DMSO-*d*_6_) δ: 1.22 (s, 2H, CH_2_ methylene), 6.89–8.59 (m, 4H, ArH), 9.00 (s, 1H, coumarin), 11.23 (s, 1H, OH exchangeable with D_2_O). IR (KBr) cm^−^^1^: 3384 (OH), 1720 (C=O coumarin), 1708 (C=O acid), 1627 (C=N), 1610 (C=C), 1223 (C=S). MS *m/z*: 300 ([M]^+^) (0.96), 257 (13.01), 256 (16.03), 225 (11.13), 213 (12.16), 185 (10.60), 150 (21.69), 149 (17.38), 136 (18.33), 119 (15.30), 111 (14.99), 79 (12.65), 72 (13.48), 70 (37.28), 68 (14.13), 67 (21.49), 69 (100). Anal. Calcd. for C_14_H_8_N_2_O_4_S: C, 56.00; H, 2.69; N, 9.33; S, 10.68. Found: C, 55.98; H, 2.70; N, 9.32; S, 10.67.

*6-(3-Oxo-3H-Benzo[f]chromen-2-yl)-2-thioxo-2,5-dihydropyrimidine-4-carboxy-lic Acid* (**10b**). Brown crystals; yield (3.08 g, 88%); m.p. 249–251 °C; DMF/H_2_O (3:1). ^1^H-NMR (DMSO-*d*_6_) *δ*: 1.23 (s, 2H), 6.90–8.05 (m, 6H), 8.75 (s, 1H), 9.78 (s, 1H). IR (KBr) cm^−1^: 3368, 1714, 1708, 1627, 1213. MS *m/z*: 352 ([M + 2]^+^), 322 (10.21), 310 (8.57), 287 (15.06), 212 (22.06), 197 (21.27), 186 (17.49), 170 (15.20), 155 (13.28), 98 (99.00), 72 (25.98), 65 (100), 56 (46.47). Anal. Calcd. for C_18_H_10_N_2_O_4_S: C, 61.71; H, 2.88; N, 8.00; S, 9.15. Found: C, 61.80; H, 2.87; N, 7.89; S, 9.14.

*2-Imino-6-(2-oxo-2H-chromen-3-yl)-2,5-dihydropyrimidine-4-carboxylic Acid* (**11a**). Deep brown crystals; yield (1.88 g, 66.4%); m.p. 228–231 °C; dioxane/H_2_O (3:1); ^1^H-NMR (DMSO-*d*_6_) δ: 1.23 (s, 2H, CH_2_ methylene), 6.90–8.05 (m, 6H, ArH), 8.75 (s, 1H, coumarin), 9.78 (s, 1H, OH, exchangeable with D_2_O). IR (KBr) cm^−1^: 3368 (OH), 1714(C=O coumarin), 1708 (C=O acid), 1627 (C=N), 1213 (C=S). MS *m/z*: 352 ([M + 2]^+^), (7.49), 322 (10.21), 310 (8.57), 287 (15.06), 212 (22.06), 197 (21.27), 186 (17.49), 170 (15.20), 155 (13.28), 98 (99.00), 72 (25.98), 65 (100), 56 (46.47). Anal. Calcd. for C_14_H_9_N_3_O_4_: C, 59.37; H, 3.20; N, 14.84. Found: C, 59.35; H, 3.21; N, 14.85.

*2-Imino-6-(3-oxo-3H-benzo[f]chromen-2-yl)-2,5-dihydropyrimidine-4-carboxylic Acid* (**11b**). Deep brown crystals; yield (2.48 g, 74.6%); m.p. 155–157 °C; DMF. ^1^H-NMR (DMSO-*d*_6_) δ: 1.21 (s, 2H, CH_2_), 2.46 (s, 1H, NH, D_2_O exchangeable), 7.17–8.21 (m, 8H, 7Ar-H, 1H coumarin). IR (KBr) cm^−1^: 3418 (OH), 3063 (NH), 1728 (C=O coumarin), 1709 (C=O acid). MS *m/z*: 335 ([M + 2]^+^), (0.95), 196 (46.29), 198 (38.27), 139 (46.56), 128 (38.16), 116 (19.92), 74 (31.27), 63 (100), 58 (70.77). Anal. Calcd. for C_18_H_11_N_3_O_4_: C, 64.86; H, 3.33; N, 12.61. Found: C, 64.80; H, 3.29; N, 12.54.

*6-(2-Oxo-2H-chromen-3-yl)-2-thioxo-2,5-dihydropyridine-3-carbonitrile* (**12a**). Brown crystals; yield (1.67 g, 59.8%); m.p. > 300 °C. ^1^H-NMR (DMSO-*d*_6_) δ: 1.27 (d, 2H, CH_2_), 6.22–7.94 (m, 6H, 4ArH, =CH, 1H, coumarin). IR (KBr) cm^−1^: 2209 (C≡N), 1713 (C=O). MS *m/z*: 280 ([M]^+^) (2.14), 167 (33.41), 149 (100), 71 (30.33). Anal. Calcd. for C_15_H_8_N_2_O_2_S: C, 64.27; H, 2.88; N, 9.99; S, 11.44. Found: C, 64.30; H, 2.87; N, 10.02; S, 11.43.

*6-(3-Oxo-3H-benzo[f]chromen-2-yl)-2-thioxo-2,5-dihydropyridine-3-carbonitrile* (**12b**). Brown crystals; yield (1.49 g, 45.2%); m.p. > 300 °C; DMF/H_2_O (3:1). ^1^H-NMR (DMSO-*d*_6_) *δ*: 1.20 (d, 2H, CH_2_), 6.81–7.95 (m, 8H, 6ArH, =CH, 1H, coumarin). IR (KBr) cm^−1^: 2207 (C≡N), 1705 (C=O). MS *m/z*: 332 ([M + 2]^+^) (2.97), 211 (13.22), 139 (20.64), 57 (92.51), 55 (100). Anal. Calcd. for C_19_H_10_N_2_O_2_S: C, 69.08; H, 3.05; N, 8.48; S, 9.71. Found: C, 69.15; H, 3.03; N, 8.46; S, 9.70.

#### 3.2.7. General Procedure for the Synthesis of Compounds **13a**,**b**

A mixture of compound **3** and/or **4** (0.01 mol), and sodium azide (0.65 g, 0.01 mol) in DMF (30 mL) was refluxed for 3 h, The mixture was poured onto iced water and the solid obtained was filtered off, dried and recrystallized from the proper solvent to give compounds **13a** and **13b**, respectively.

*3-(1,2,3-Triazin-4-yl)-2H-chromen-2-one* (**13a**). Pale brown crystals; yield (2.00 g, 89.2%); m.p. 270–272 °C; dioxane/H_2_O (3:1). ^1^H-NMR (DMSO-*d*_6_) δ: 6.60–8.20 (m, 6H, 4ArH, 2=CH), 8.89 (s, 1H, coumarin). IR (KBr) cm^−1^: 1718 (C=O), 1631 (C=N). MS *m/z*: 227 ([M + 2]^+^) (16.37), 200 (24.66), 199 (25.61), 189 (22.85), 175 (21.17), 157 (20.19), 147 (23.03), 145 (28.23), 133 (25.42), 124 (19.35), 110 (24.98), 103 (27.33), 92 (23.60), 85 (19.39), 74 (13.75), 73 (100). Anal. Calcd. for C_12_H_7_N_3_O_2_: C, 64.00; H, 3.13; N, 18.66. Found: C, 64.16; H, 3.14; N, 18.69.

*2-(1,2,3-Triazin-4-yl)-3H-benzo[f]chromen-3-one* (**13b**). Brown crystals; yield (1.72 g, 62.7%); m.p. > 300 °C; DMF/H_2_O (3:1). ^1^H-NMR (DMSO-*d*_6_) δ: 6.42–8.16 (m, 8H, 6ArH, 2=CH), 8.22 (s, 1H, coumarin). IR (KBr) cm^−1^:1707 (C=O), 1624(C=N). MS *m/z*: 275 ([M]^+^) (0.09), 196 (0.98), 98 (98.39), 80 (100), 66 (12.96), 64 (97.95). Anal. Calcd. for C_16_H_9_N_3_O_2_: C, 69.81; H, 3.30; N, 15.27. Found: C, 69.87; H, 3.29; N, 15.24.

#### 3.2.8. General Procedure for the Synthesis of Compounds **14a,b**

A mixture of compound **3** and/or **4** (0.01 mol) and Lawesson’s reagent (4.0 g, 0.01 mol) was refluxed in acetonitrile (30 mL) for 28 h. The reaction mixture was filtered while hot and the filtrate was left to cool at room temperature. The obtained solid was filtered off, dried and recrystallized from the proper solvent to give compounds **14a** and **14b** respectively.

*3-(5-Hydroxythiophen-2-yl)-2H-thiochromen-2-one* (**14a**) Pale brown crystals; yield (1.99 g, 76.9%); m.p. 250–253 °C; ethanol/DMF (2:1). ^1^H-NMR (DMSO-*d*_6_) δ: 6.91 (d, 1H, =CH), 7.14 (s, 1H, OH D_2_O exchangeable), 7.54–7.61 (m, 6H, 4ArH, =CH, 1H coumarin). IR (KBr) cm^−1^ 3557 (OH), 1724 (C=O), 1602 (C=C). MS *m/z*: 261 ([M + 1]^+^) (0.13), 188 (100), 108 (62.03), 94 (19.60), 92 (16.20), 77 (39.43), 62 (33.50), 46 (28.69). Anal. Calcd. for C_13_H_8_O_2_S_2_: C, 59.98; H, 3.10; S, 24.63. Found: C, 59.91; H, 3.13; S, 24.66.

*2-(5-Hydroxythiophen-2-yl)-3H-benzo[f]thiochromen-3-one* (**14b**). Brown crystals; yield (1.76 g, 56.9%); m.p. > 300 °C; DMF/H_2_O (3:1). ^1^H-NMR (DMSO-*d*_6_) δ: 6.20 (s, 1H, OH, D_2_O exchangeable), 6.93 (d, 1H, *J* = 3.00, =CH), 6.96 (d, 1H, *J* = 3.00, =CH), 7.37–8.64 (m, 6H, ArH), 9.28 (s, 1H, coumarin). IR (KBr) cm^−1^ 3428 (OH), 1724 (C=O), 1630 (C=C). MS *m/z* (%): 310 ([M]^+^) (8.69), 282 (13.31), 172 (12.06), 128 (11.51), 86 (22.08), 84 (48.17), 70 (25.06), 57 (100). Anal. Calcd. for C_17_H_10_O_2_S_2_: C, 65.78; H, 3.25; S, 20.66. Found: C, 59.95; H, 3.23; S, 20.65.

#### 3.2.9. General Procedure for the Synthesis of Compounds **(15**–**17)a,b**

A mixture of compound **3** and/or **4** (0.01 mol), diethyl malonate, ethyl cyanoacetate and malononitrile (0.01 mol) in dry acetone (30 mL) was refluxed on a water bath for 24 h in the presence of K_2_CO_3_ (2.76 g, 0.02 mole) and tetrabutyl-*n*-ammonium bromide (0.64 g, 0.002 mol). The excess solvent was evaporated and the reaction mixture was poured into water. The separated solid was filtered off, dried and recrystallized from the suitable solvent to give compounds (**15**–**17**)**a**,**b**.

*Ethyl 2-Oxo-5-(2-oxo-2H-chromen-3-yl)-3,4-dihydro-2H-pyran-3-carboxylate* (**15a**). Brown crystals; yield (2.12 g, 67.8%); m.p. > 300 °C; acetic acid/water (2:1). ^1^H-NMR (DMSO-*d*_6_) δ: 0.93 (t, 3H, CH_3_), 1.22–1.35 (m, 2H, CH_2_), 1.50–1.62 (m, 1H, CH), 3.16–3.18 (q, 2H, OCH_2_), 6.50–8.16 (m, 5H, 4ArH, =CH), 8.59 (s, 1H, coumarin). IR (KBr) cm^−1^ 1715 (C=O). Anal. Calcd. for C_17_H_14_O_6_: C, 64.97; H, 4.49. Found: C, 64.90; H, 4.47.

*Ethyl 2-Oxo-5-(3-oxo-3H-benzo[f]chromen-2-yl)-3,4-dihydro-2H-pyran-3-carboxylate* (**15b**). Brown crystals; yield (2.35 g, 64.8%); m.p. > 300 °C; toluene. ^1^H-NMR (DMSO-*d*_6_) δ: 0.93 (t, 3H, CH_3_, *J* = 7.5 Hz), 1.23–1.34 (m, 2H, CH_2_), 1.50–1.63 (m, 1H, CH), 3.10–3.18 (q, 2H, OCH_2_, *J* = 7.5 Hz), 7.15 (t, 1H, =CH), 7.12–8.08 (m, 6H, ArH), 8.53 (s, 1H, coumarin). IR (KBr) cm^−1^ 1716, broad 1625 (C=O). Anal. Calcd. for C_21_H_16_O_6_: C, 69.23; H, 4.43. Found: C, 69.25; H, 4.41.

*Ethyl 2-amino-6-(2-oxo-2H-chromen-3-yl)-4H-pyran-3-carboxylate* (**16a**). Brown crystals; yield (2.31 g, 74%); m.p. > 300 °C; ethanol/toluene (2:1). ^1^H-NMR (DMSO-*d*_6_) δ: 1.05 (t, 3H, CH_3_, *J* = 7.2 Hz), 2.72–2.74 (m, 2H, CH_2_), 3.14–3.18 (q, 2H, OCH2, *J* = 7.2 Hz), 4.00 (s, 2H, NH_2_, D_2_O exchangeable), 6.96–7.90 (m, 6H, 4ArH, =CH, CH coumarin). IR (KBr) cm^−1^: 3418, 3228 (NH_2_), 1712 (C=O). Anal. Calcd. for C_17_H_15_NO_5_: C, 65.17; H, 4.83; N, 4.47. Found: C, 65.10; H, 4.81; N, 4.48.

*Ethyl 2-Amino-6-(3-oxo-3H-benzo[f]chromen-2-yl)-4H-pyran-3-carboxylate* (**16b**). Brown crystals; yield (2.61 g, 72%); m.p. > 300 °C; acetic acid/water (2:1). ^1^H-NMR (DMSO-*d*_6_) δ: 0.92 (t, 3H, CH_3_, *J* = 7.2 Hz), 1.23–1.33 (m, 2H, CH_2_), 2.57 (s, 2H, NH_2_, D_2_O exchangeable), 3.12–3.18 (q, 2H, OCH_2_, *J* = 7.2 Hz), 6.58 (t, 1H, =CH), 7.00–8.50 (m, 6H, ArH), 8.95 (s, 1H, coumarin). IR (KBr) cm^−1^: 3409, 3242 (NH_2_), 1712 (C=O). Anal. Calcd. for C_21_H_17_NO_5_: C, 69.41; H, 4.72; N, 3.85. Found: C, 69.46; H, 4.71; N, 3.86.

*2-(Dicyanomethyl)-4-oxo-4-(2-Oxo-2H-chromen-3-yl)butanoic Acid* (**17a**). Brown crystals; yield (2.41 g, 78%); m.p. > 300 °C; ethanol. ^1^H-NMR (DMSO-*d*_6_) δ: 2.24–2.28 (m, 1H, CH), 2.72–2.76 (m, 2H,CH_2_), 2.83–2.88 (m, 1H, CH), 7.41–8.44 (m, 4H, ArH), 8.56 (s, 1H, coumarin), 12.22 (s, 1H, OH, D_2_O exchangeable). IR (KBr) cm^−1^: 3434 (OH), 2209 (C≡N), 1708, 1646 (C=O). Anal. Calcd. for C_16_H_10_N_2_O_5_: C, 61.94; H, 3.25; N, 9.03. Found: C, 61.99; H, 3.24; N, 9.13.

*2-(Dicyanomethyl)-4-oxo-4-(3-oxo-3H-benzo[f]chromen-2-yl)butanoic Acid* (**17b**). Brown crystals; yield (2.59 g, 72.2%); m.p. > 300 °C; DMF. ^1^H-NMR (DMSO-*d*_6_) δ: 2.63–2.67 (m, 1H, CH), 2.72–2.79 (m, 2H, CH_2_), 2.88–2.91 (m, 1H, CH), 7.04–8.64 (m, 4H, ArH), 8.93 (s, 1H, coumarin), 12.56 (s, 1H, OH, D_2_O exchangeable). IR (KBr) cm^−1^: 3434 (OH), 2210 (C≡N), 1713, 1632 (C=O). Anal. Calcd. for C_20_H_12_N_2_O_5_: C, 66.67; H, 3.36; N, 7.77. Found: C, 66.64; H, 3.37; N, 7.79.

#### 3.2.10. General Procedure for the Synthesis of Compounds **18a** and **18b**

A mixture of compounds **17a**,**b** (3.10 g, 0.01 mol), hydrazine hydrate (0.50 mL, 0.01 mol) in DMF (20 mL) was refluxed for 3 h, cool, poured on water, The solid that separated was filtered off, dried and recrystallized from the proper solvent to give compounds **18a** and **18b** respectively.

*2-(3-Oxo-6-(2-oxo-2H-chromen-3-yl)-2,3,4,5-tetrahydropyridazin-4-yl)malononitrile* (**18a**). Brown crystals; yield (2.40 g, 83%); m.p. > 300 °C; AcOH/H_2_O (3:1). ^1^H-NMR (DMSO-*d*_6_) δ: 1.23–1.27 (m, 1H, CH), 1.90–1.95 (m, 2H, CH_2_), 2.71 (d, 1H, CH), 6.50–8.00 (m, 4H, ArH), 8.13 (s, 1H, coumarin), 8.60 (s, 1H, NH, D_2_O exchangeable). IR (KBr) cm^−1^: 3362 (NH), 2211 (C≡N), 1627 (C=O). Anal. Calcd. for C_16_H_10_N_4_O_3_: C, 62.74; H, 3.29; N, 18.29. Found: C, 62.75; H, 3.27; N, 18.28.

*2-(3-Oxo-6-(3-oxo-3H-benzo[f]chromen-2-yl)-2,3,4,5-tetrahydropyridazin-4-yl)malononitrile* (**18b**). Brown crystals; yield (2.70 g, 79.6%); m.p. > 300 °C; DMF. ^1^H-NMR (DMSO-*d*_6_) δ: 1.20–1.22 (m, 1H, CH), 1.92–1.97 (m, 2H, CH_2_), 2.21 (d, 1H, CH), 6.56–8.25 (m, 6H, ArH), 8.13 (s, 1H, coumarin), 8.54 (s, 1H, NH, D_2_O exchangeable). IR (KBr) cm^−1^: 3399 (NH), 2207 (C≡N), 1627 (C=O). Anal. Calcd. for C_20_H_12_N_4_O_3_: C, 67.41; H, 3.39; N, 15.72. Found: C, 67.42; H, 3.38; N, 15.73.

#### 3.2.11. Synthesis of 2-(6-Oxo-3-(2-oxo-2*H*-chromen-3-yl)-5,6-dihydro-4*H*-1,2-oxazin-5-yl)malon-onitrile **19**

A mixture of compound **17** (3.10 g, 0.01 mol), and hydroxylamine hydrochloride (0.69 g, 0.01 mol) in pyridine (20 mL) was refluxed for 3 h, cool, poured onto ice/HCl, The solid that separated was filtered off, dried and recrystallized from ethanol/benzene to give compound **19** as brown crystals; yield (2.01 g, 65.5%); m.p. > 300 °C; ethanol/benzene (2:1). ^1^H-NMR (DMSO-*d*_6_) δ*:* 1.22–1.25 (d, 2H, CH_2_), 2.26–2.30 (m, 1H, CH), 2.72 (d, 1H, CH), 7.05–8.34 (m, 4H, ArH), 8.77 (s, 1H, coumarin). IR (KBr) cm^−1^: 2209 (C≡N), 1713, 1627 (C=O). Anal. Calcd. for C_16_H_9_N_3_O_4_: C, 62.54; H, 2.95; N, 13.68; Found: C, 62.51; H, 2.93; N, 13.66.

### 3.3. Pharmacological Activity

#### 3.3.1. Cytotoxicity Assay

The cytotoxic activity of twenty two compounds was tested against four human tumor cell lines namely: hepatocellular carcinoma (liver) HePG2, colon cancer HCT-116, human (prostate) cancer PC3 and mammary gland (breast) MCF-7. The cell lines were obtained from ATCC via the Holding company for biological products and vaccines (VACSERA, Cairo, Egypt). 5-Fluorouracil was used as a standard anticancer drug for comparison. The reagents used were RPMI-1640 medium, MTT, DMSO and 5-fluorouracil (Sigma Co., St. Louis, MO, USA), and fetal bovine serum (GIBCO, Paisely, UK).

##### MTT Assay

The different cell lines [54,55] mentioned above were used to determine the inhibitory effects of compounds on cell growth using the MTT assay. This colorimetric assay is based on the conversion of the yellow tetrazolium bromide (MTT) to a purple formazan derivative by mitochondrial succinate dehydrogenase in viable cells. The cells were cultured in RPMI-1640 medium with 10% fetal bovine serum. Antibiotics added were 100 units/mL penicillin and 100 µg/mL streptomycin at 37 °C in a 5% CO_2_ incubator. The cells lines were seeded [56] in a 96-well plate at a density of 1.0 × 10^4^ cells/well at 37 °C for 48 h under 5% CO_2_. After incubation the cells were treated with different concentration of compounds and incubated for 24 h. After 24 h of drug treatment, 20 µL of MTT solution at 5 mg/mL was added and incubated for 4 h. Dimethyl sulfoxide (DMSO) in volume of 100 µL is added into each well to dissolve the purple formazan formed. The colorimetric assay is measured and recorded at absorbance of 570 nm using a plate reader (EXL 800, BioTech, Winooski, VT, USA). The relative cell viability in percentage was calculated as (A_570_ of treated samples/A_570_ of untreated sample) × 100.

#### 3.3.2. Antioxidant Assay

##### ABTS Method

For each of the investigated compounds [57,58,59] of ABTS solution (60 µM, 2 mL) was added to MnO_2_ suspension (25 mg/mL, 3 mL), all prepared in aqueous phosphate buffer solution (pH 7, 0.1 M, 5 mL). The mixture was shaken, centrifuged, filtered and the absorbance of the resulting green blue solution (ABTS radical solution) at 734 nm was adjusted to approx. ca. 0.5. Then, asolution (50 µL of (2 mM) of the tested compound in spectroscopic grade MeOH/phosphate buffer (1:1) was added. The absorbance was measured and the reduction in color intensity was expressed as inhibition percentage. L–ascorbic acid was used as standard antioxidant (positive control). Blank sample was run without ABTS and using MeOH/phosphate buffer (1:1) instead of the tested compounds. Negative control was run with ABTS and MeOH/phosphate buffer (1:1) only.

##### Bleomycin—Dependent DNA Damage Assay

To the reaction mixtures [60,61] in a final volume of 1.0 mL, the following reagents were added: DNA (0.2 mg/mL), bleomycin sulfate (0.05 mg/mL), FeCl_3_ (0.025 mM), magnesium chloride (5 mM), KH_2_PO_4–_KOH buffer pH 7.0 (30 mM), and ascorbic acid (0.24 mM) or the test fractions diluted in MeOH to give a concentration of (0.1 mg/mL). The reaction mixtures were incubated in a water bath at 37 °C for 1 h. At the end of the incubation period, 0.1 mL of ethylenediaminetetraacetic acid (EDTA) (0.1 M) was added to stop the reaction (the iron-EDTA complex is unreactive in the bleomycin assay). DNA damage was assessed by adding 1 mL 1% (*w/v*) thiobarbituric acid (TBA) and 1 mL of 25% (*v/v*) hydrochloric acid followed by heating in a water-bath maintained at 80 °C for 15 min. The chromogenic formed was extracted into 1-butanol, and the absorbance was measured at 532 nm.

## 4. Conclusions

The objective of the present study was to synthesize the coumarin scaffold-based compounds and evaluate their cytotoxicity, antioxidant and bleomycin dependent DNA damage protection activities. The tested compounds showed very strong to non-cytotoxic activity against four anticancer cell lines. The best results were observed for compounds **5a**, **7a**,**b**, **9a**,**b**, **13b**, **15a,b**, **16a** and **18a**. Compound **15b** showed activity approximately equal to that of 5-FU as a standard against PC3 and compound **7b** showed higher activity than the 5-FU against HCT-116.

## Figures and Tables

**Table 1:** Cytotoxicity (IC_50_) of the tested compounds on different cell lines.

Comp. No.	IC_50_ (μg/mL) ^a^			
	HePG2	HCT-116	PC3	MCF-7
3	41.2 ± 3.06	36.5 ± 02.54	42.0 ± 3.24	45.8 ± 3.40
**4**	51.2 ± 3.62	33.6 ± 2.64	48.9 ± 2.91	39.7 ± 2.35
**5a**	12.5 ± 0.69	12.8 ± 1.03	16.2 ± 1.56	15.7 ± 1.24
**5b**	27.6 ± 1.87	21.8 ± 2.10	34.0 ± 2.65	15.8 ± 1.08
**7a**	10.8 ± 0.88	10.4 ± 0.94	9.6 ± 0.382	10.6 ± 0.92
**7b**	9.3 ± 0.58	4.8 ± 0.18	11.1 ± 1.13	7.8 ± 0.67
**9a**	13.1 ± 0.95	9.4 ± 0.97	14.5 ± 1.30	12.0 ± 1.14
**9b**	11.0 ± 0.98	11.4 ± 0.87	18.0 ± 1.96	20.4 ± 1.56
**10a**	35.7 ± 2.54	46.3 ± 2.35	51.6 ± 3.61	29.9 ± 1.97
**10b**	63.5 ± 3.94	56.4 ± 3.35	83.8 ± 3.58	64.4 ± 3.84
**11a**	73.0 ± 4.35	78.2 ± 3.98	96.8 ± 4.87	81.5 ± 4.21
**11b**	22.6 ± 1.36	19.8 ± 1.63	25.5 ± 1.74	5.6 ± 0.43
**13a**	52.9 ± 3.62	65.1 ± 4.11	76.9 ± 4.65	58.3 ± 3.63
**13b**	17.4 ± 1.25	14.4 ± 1.01	10.8 ± 0.79	29.6 ± 1.87
**15a**	17.6 ± 1.05	20.0 ± 1.44	13.7 ± 0.94	17.6 ± 1.37
**15b**	8.2 ± 0.45	9.7 ± 0.84	8.7 ± 0.45	14.1 ± 1.21
**16a**	25.6 ± 1.67	15.3 ± 1.13	13.4 ± 0.96	19.9 ± 2.14
**16b**	26.2 ± 1.13	29.6 ± 2.25	35.4 ± 2.13	40.2 ± 2.64
**17a**	90.6 ± 6.57	71.1 ± 4.82	87.4 ± 5.14	86.7 ± 6.15
**17b**	52.7 ± 3.41	44.7 ± 3.12	59.8 ± 2.35	45.9 ± 2.89
**18a**	19.7 ± 1.20	13.8 ± 0.89	16.1 ± 1.08	13.2 ± 0.76
**18b**	60.1 ± 3.24	72.6 ± 3.86	82.2 ± 4.32	62.5 ± 4.35
**19**	25.2 ± 2.10	22.4 ± 1.37	28.8 ± 2.67	25.8 ± 1.72
5-FU	7.9 ± 0.12	5.3 ± 0.14	8.3 ± 0.25	5.4 ± 0.21

^a^ IC_50_ (μg/mL): 1–10 (very strong), 11–20 (strong), 21–50 (moderate), 51–100 (weak), above 100 (non-cytotoxic).

**Table 2:** Antioxidant activity and bleomycin-dependent DNA damage for the tested compounds ^a^.

Comp. No.	Antioxidant Activity (ABTS Method)		Bleomycin Dependent DNA Damage
	Absorbance	Inhibition (%)	
**3**	0.233	51.6	0.095
**4**	0.134	72.9	0.114
**5a**	0.145	69.9	0.107
**5b**	0.078	84.4	0.089
**7a**	0.119	75.3	0.089
**7b**	0.076	84.6	0.083
**9a**	0.150	68.9	0.099
**9b**	0.076	84.6	0.096
**10a**	0.236	51.0	0.126
**10b**	0.217	56.2	0.131
**11a**	0.248	49.9	0.142
**11b**	0.077	84.4	0.072
**13a**	0.244	49.4	0.145
**13b**	0.076	84.6	0.105
**15a**	0.201	58.3	0.094
**15b**	0.118	75.5	0.076
**16a**	0.195	59.5	0.089
**16b**	0.232	51.9	0.106
**17a**	0.290	39.8	0.18
**17b**	0.152	69.3	0.127
**18a**	0.164	66.0	0.081
**18b**	0.274	43.1	0.125
**19**	0.213	55.8	0.124
Ascorbic acid	0.053	89.0	0.073

^a^ All experiments were performed three times. The data are expressed as the mean-standard error of the mean (S.E.M.).

## Supplementary Materials

Supplementary materials can be accessed at: http://www.mdpi.com/1420-3049/21/2/249/s1.
