# Supplementary material for: Synthesis and Characterization of Some New Coumarins with in Vitro Antitumor and Antioxidant Activity and High Protective Effects against DNA Damage
Source: Molecules. 2016 Feb 22;21(2):249. doi: 10.3390/molecules21020249 (PMC6274385; doi:10.3390/molecules21020249)
Supplement: Supplementary file 1 [file molecules-21-00249-s001.pdf]

Supplementary Materials: Synthesis and Characterization of Some New Coumarins with *In Vitro* Antitumor and Antioxidant Activity and High Protective Effects against DNA Damage

Mounir A. I. Salem [1,†], Magda I. Marzouk [1,†,*] and Azza M. El-Kazak [2,†]

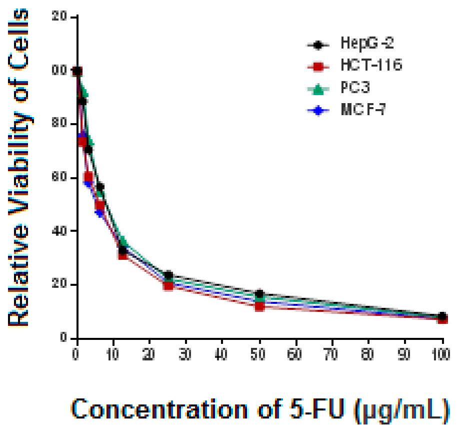

Figure S1. Relative viability of cells (%) against concentration of 5-FU.

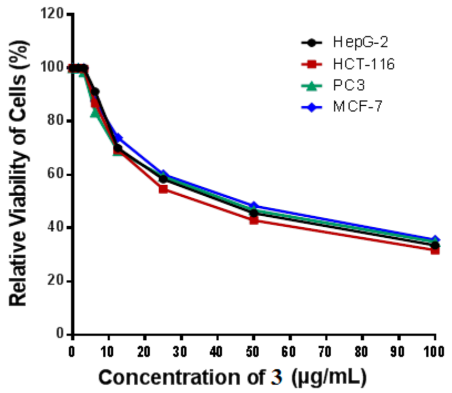

Figure S2. Relative viability of cells (%) against concentration of compound **3**.

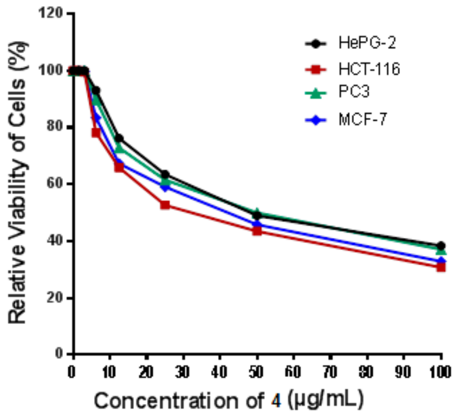

Figure S3. Relative viability of cells (%) against concentration of compound **4**.

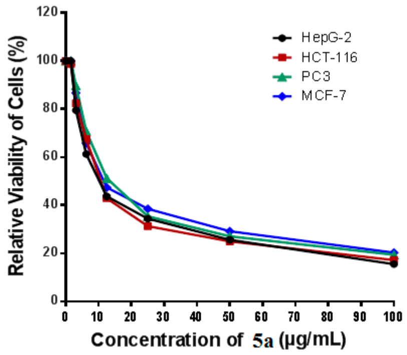

Figure S4. Relative viability of cells (%) against concentration of compound **5a**.

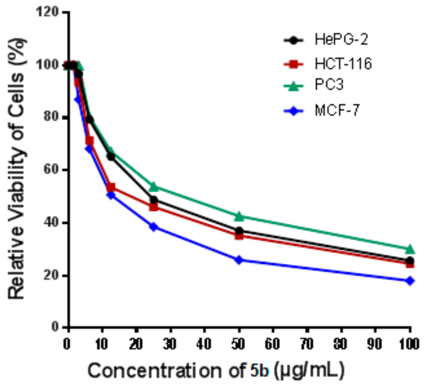

Figure S5. Relative viability of cells (%) against concentration of compound **5b**.

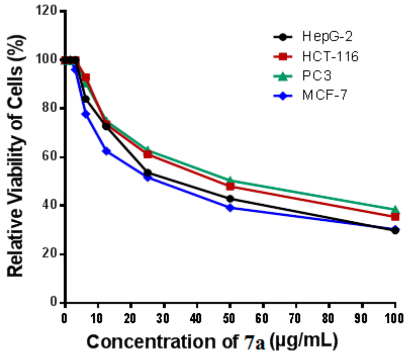

Figure S6. Relative viability of cells (%) against concentration of compound **7a**.

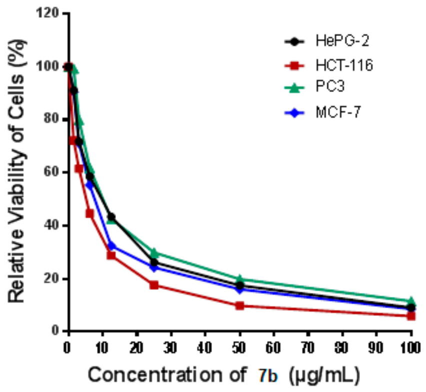

Figure S7. Relative viability of cells (%) against concentration of compound **7b**.

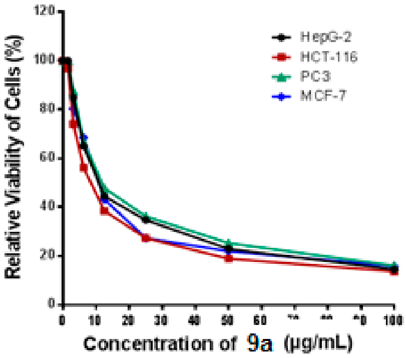

Figure S8. Relative viability of cells (%) against concentration of compound **9a**.

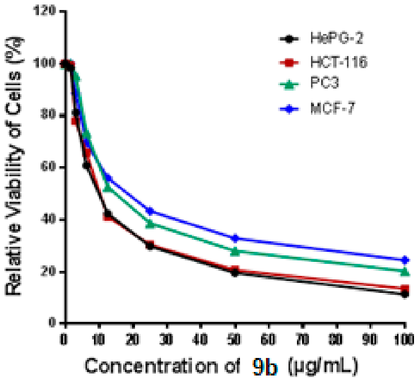

Figure S9. Relative viability of cells (%) against concentration of compound **9b**.

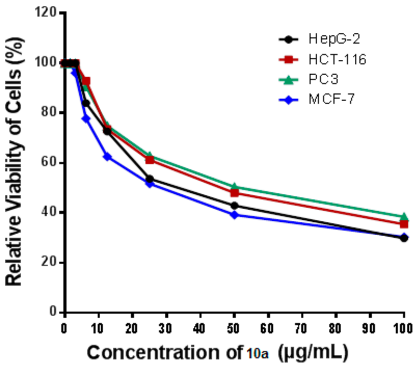

Figure S10. Relative viability of cells (%) against concentration of compound **10a**.

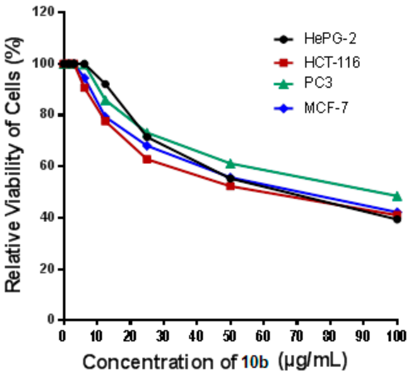

Figure S11. Relative viability of cells (%) against concentration of compound **10b**.

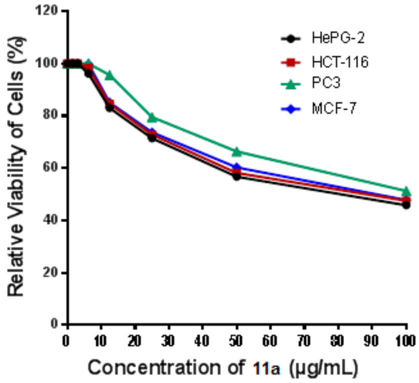

Figure S12. Relative viability of cells (%) against concentration of compound **11a**.

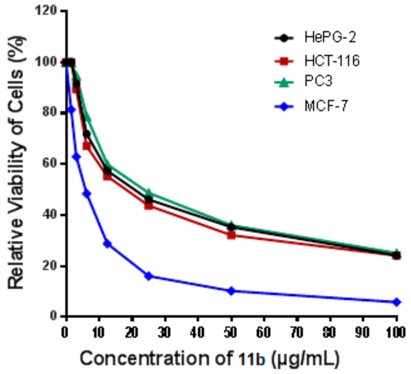

Figure S13. Relative viability of cells (%) against concentration of compound **11b**.

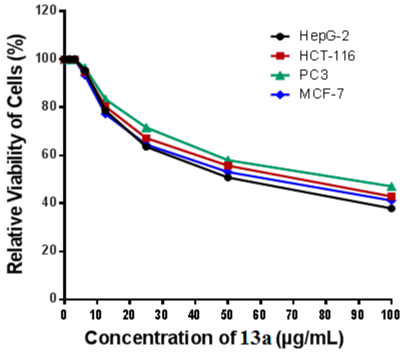

Figure S14. Relative viability of cells (%) against concentration of compound **13a**.

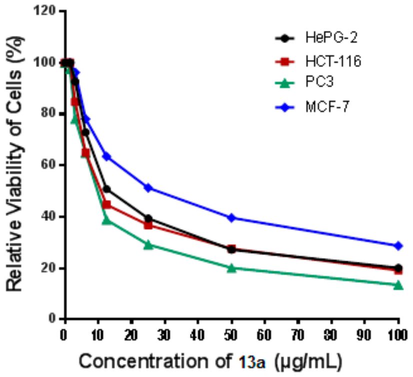

Figure S15. Relative viability of cells (%) against concentration of compound **13b**.

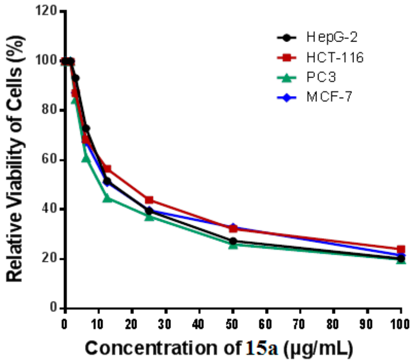

Figure S16. Relative viability of cells (%) against concentration of compound **15a**.

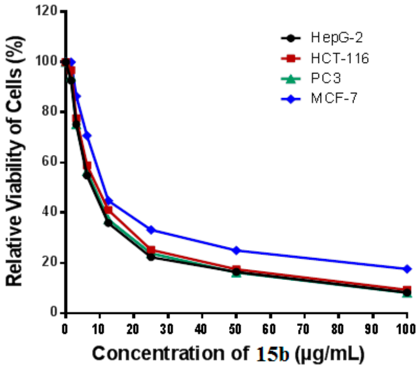

Figure S17. Relative viability of cells (%) against concentration of compound **15b**.

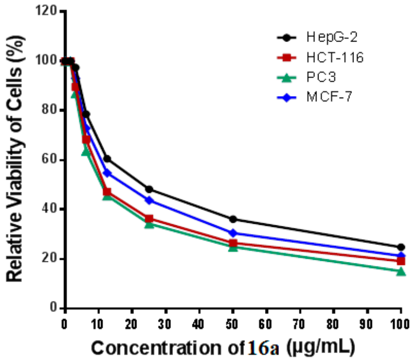

Figure S18. Relative viability of cells (%) against concentration of compound **16a**.

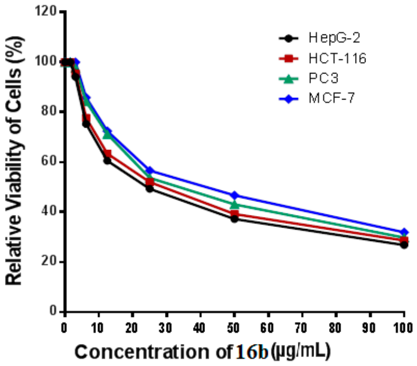

Figure S19. Relative viability of cells (%) against concentration of compound**16b**.

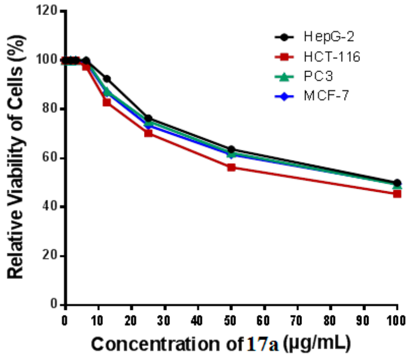

Figure S20. Relative viability of cells (%) against concentration of compound **17a**.

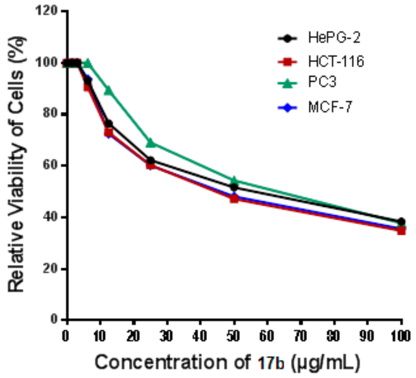

Figure S21. Relative viability of cells (%) against concentration of compound **17b**.

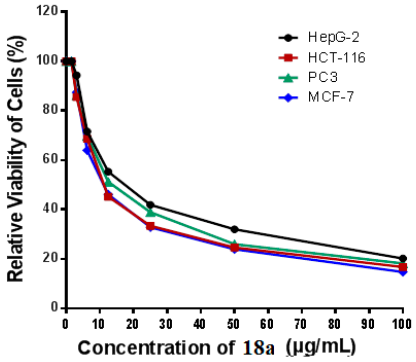

Figure S22. Relative viability of cells (%) against concentration of compound **18a**.

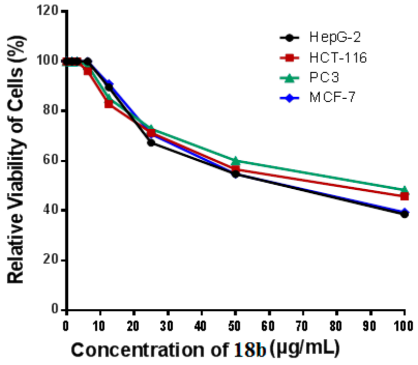

Figure S23. Relative viability of cells (%) against concentration of compound **18b**.

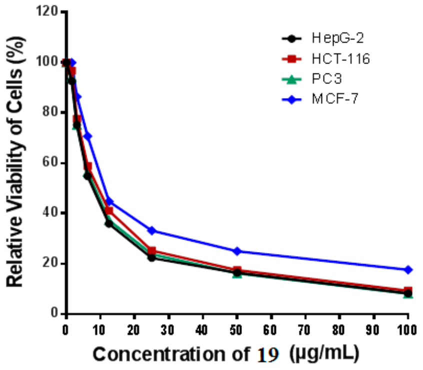

Figure S24. Relative viability of cells (%) against concentration of compound **19**.

Table S1. Relative viability of cells (%) for 5-FU and compounds **3**, **4**, **5a**,**b**, **7a**,**b** and **(9–10)a**,**b**.

Compounds	Conc. (µg/mL)	HepG-2	HCT-116	PC3	MCF-7
5-FU	100 µg/mL	8.6	7.4	8.1	7.6
	50 µg/mL	17.1	12.1	15.8	14.1
	25 µg/mL	24.0	19.8	22.5	21.0
	12.5 µg/mL	33.1	31.4	36.7	34.5
	6.25 µg/mL	56.8	49.9	55.2	47.3
	3.125 µg/mL	70.6	60.5	74.1	58.2
	1.56 µg/mL	88.7	73.6	92.5	76.0
**3**	100 µg/mL	33.5	31.7	34.8	35.6
	50 µg/mL	45.6	42.9	46.7	48.3
	25 µg/mL	58.3	54.6	59.3	60.1
	12.5 µg/mL	70.0	69.3	68.9	73.9
	6.25 µg/mL	91.2	86.8	83.4	87.6
	3.125 µg/mL	100	99.7	98.5	100
	1.56 µg/mL	100	100	100	100
**4**	100 µg/mL	38.4	30.8	37.1	32.9
	50 µg/mL	49.0	43.5	50.0	45.8
	25 µg/mL	63.5	52.7	61.6	59.1
	12.5 µg/mL	76.2	65.9	72.9	67.3
	6.25 µg/mL	93.1	78.2	89.8	83.4
	3.125 µg/mL	100	99.5	100	99.7
	1.56 µg/mL	100	100	100	100
**5a**	100 µg/mL	15.5	17.2	19.4	20.3
	50 µg/mL	25.6	25.0	27.1	29.2
	25 µg/mL	34.4	31.3	35.4	38.5
	12.5 µg/mL	43.7	42.9	51.2	47.4
	6.25 µg/mL	61.3	67.5	70.8	65.8
	3.125 µg/mL	79.5	82.4	89.7	86.7
	1.56 µg/mL	100	98.7	100	100
**5b**	100 µg/mL	25.7	24.5	30.1	18.0
	50 µg/mL	37.1	35.2	42.6	25.9
	25 µg/mL	48.9	46.1	53.9	38.5
	12.5 µg/mL	65.2	53.6	67.2	50.7
	6.25 µg/mL	79.3	71.4	80.4	68.2
	3.125 µg/mL	96.8	93.5	99.9	87.0
	1.56 µg/mL	100	100	100	100

Table S1. *Cont.*

Compounds	Conc. (μg/mL)	HepG-2	HCT-116	PC3	MCF-7
**7a**	100 μg/mL	11.4	13.1	9.1	14.5
	50 μg/mL	18.8	18.9	16.8	20.8
	25 μg/mL	28.7	27.0	26.3	25.4
	12.5 μg/mL	42.1	37.4	38.9	38.9
	6.25 μg/mL	61.5	66.9	59.6	67.7
	3.125 μg/mL	80.2	76.6	78.2	75.0
	1.56 μg/mL	98.3	97.2	96.7	97.3
**7b**	100 μg/mL	9.1	5.9	11.5	8.6
	50 μg/mL	17.5	9.8	19.8	16.0
	25 μg/mL	26.2	17.6	29.9	24.3
	12.5 μg/mL	43.4	28.7	42.6	32.4
	6.25 μg/mL	58.6	44.6	61.8	55.3
	3.125 μg/mL	71.8	61.5	80.0	71.1
	1.56 μg/mL	91.1	72.2	99.3	90.5
**9a**	100 μg/mL	14.5	13.7	16.0	15.8
	50 μg/mL	22.9	18.9	25.3	22.0
	25 μg/mL	34.7	27.3	36.2	27.3
	12.5 μg/mL	44.5	38.5	47.8	43.2
	6.25 μg/mL	65.0	56.2	66.7	68.5
	3.125 μg/mL	84.8	74.1	87.5	80.4
	1.56 μg/mL	100	96.8	100	98.6
**9b**	100 μg/mL	11.4	13.5	20.3	24.5
	50 μg/mL	19.6	20.7	28.0	32.9
	25 μg/mL	29.8	30.4	38.7	43.3
	12.5 μg/mL	42.5	41.2	52.6	56.1
	6.25 μg/mL	60.9	65.8	73.5	69.7
	3.125 μg/mL	81.2	78.0	95.4	88.6
	1.56 μg/mL	98.3	99.1	100	100
**10a**	100 μg/mL	29.8	35.4	38.4	30.3
	50 μg/mL	42.9	48.0	50.4	39.2
	25 μg/mL	53.7	61.2	62.8	51.7
	12.5 μg/mL	72.6	73.5	74.9	62.5
	6.25 μg/mL	84.0	92.9	90.7	77.8
	3.125 μg/mL	100	100	100	96.1
	1.56 μg/mL	100	100	100	100
**10b**	100 μg/mL	39.4	41.0	48.5	42.2
	50 μg/mL	55.3	52.3	61.2	55.7
	25 μg/mL	71.5	62.8	73.2	68.1
	12.5 μg/mL	92.1	77.6	85.9	79.3
	6.25 μg/mL	100	90.7	99.7	94.5
	3.125 μg/mL	100	100	100	100
	1.56 μg/mL	100	100	100	100

Table S2. Relative viability of cells (%) for 5-FU and compounds **11a,b**, **13a,b (15–18)a,b** and **19**.

Compounds	Conc. (µg/mL)	HepG-2	HCT-116	PC3	MCF-7
**11a**	100 µg/mL	45.8	47.5	51.3	47.8
	50 µg/mL	56.7	58.2	66.4	60.3
	25 µg/mL	71.4	72.8	79.5	73.7
	12.5 µg/mL	83.0	84.6	95.6	85.1
	6.25 µg/mL	96.2	98.3	100	99.8
	3.125 µg/mL	100	100	100	100
	1.56 µg/mL	100	100	100	100
	1.56 µg/mL	45.8	47.5	51.3	47.8
**11b**	100 µg/mL	24.2	24.1	25.1	5.8
	50 µg/mL	35.3	32.1	36.0	10.2
	25 µg/mL	46.1	43.7	48.7	16.1
	12.5 µg/mL	57.4	55.2	59.9	28.8
	6.25 µg/mL	71.9	67.1	78.8	48.4
	3.125 µg/mL	91.7	89.4	94.7	62.9
	1.56 µg/mL	100	100	100	81.4
	1.56 µg/mL	24.2	24.1	25.1	5.8
**13a**	100 µg/mL	37.9	42.9	47.1	41.2
	50 µg/mL	50.8	55.7	58.0	53.1
	25 µg/mL	63.6	67.2	71.6	64.5
	12.5 µg/mL	78.7	80.4	83.4	77.3
	6.25 µg/mL	95.3	94.7	96.5	93.4
	3.125 µg/mL	100	100	100	100
	1.56 µg/mL	100	100	100	100
	1.56 µg/mL	37.9	42.9	47.1	41.2
**13b**	100 µg/mL	20.1	19.2	13.5	28.7
	50 µg/mL	27.2	27.6	20.1	39.6
	25 µg/mL	39.4	36.8	29.2	51.3
	12.5 µg/mL	50.8	44.7	38.8	63.5
	6.25 µg/mL	72.9	65.0	64.9	78.1
	3.125 µg/mL	92.7	84.8	78.1	96.2
	1.56 µg/mL	100	100	97.6	100
**15a**	100 µg/mL	20.2	24.0	19.8	21.5
	50 µg/mL	27.3	32.2	25.9	32.8
	25 µg/mL	39.4	43.9	37.2	39.7
	12.5 µg/mL	51.6	56.4	44.7	51.0
	6.25 µg/mL	72.9	68.3	61.0	67.4
	3.125 µg/mL	93.2	87.1	84.6	87.3
	1.56 µg/mL	100	99.8	100	100
**15b**	100 µg/mL	8.2	9.3	8.1	17.6
	50 µg/mL	16.4	17.5	16.2	25.0
	25 µg/mL	22.3	25.2	23.8	33.2
	12.5 µg/mL	35.9	41.1	37.3	44.8
	6.25 µg/mL	54.8	58.9	56.5	70.7
	3.125 µg/mL	75.2	77.6	75.1	86.4
	1.56 µg/mL	92.6	96.8	94.2	100

Table S2. *Cont.*

Compounds	Conc. (µg/mL)	HepG-2	HCT-116	PC3	MCF-7
**16a**	100 µg/mL	24.8	19.1	15.1	21.3
	50 µg/mL	36.1	26.5	24.9	30.5
	25 µg/mL	48.2	36.4	34.3	43.7
	12.5 µg/mL	60.5	47.2	45.6	54.8
	6.25 µg/mL	78.6	68.4	63.7	72.9
	3.125 µg/mL	97.4	89.6	87.0	93.1
	1.56 µg/mL	100	100	100	100
**16b**	100 µg/mL	26.8	28.6	29.8	31.9
	50 µg/mL	37.2	39.2	43.1	46.7
	25 µg/mL	49.3	52.0	53.7	56.6
	12.5 µg/mL	60.5	63.4	71.2	72.4
	6.25 µg/mL	75.3	77.7	84.3	85.8
	3.125 µg/mL	94.1	95.5	97.6	100
	1.56 µg/mL	26.8	28.6	29.8	31.9
**17a**	100 µg/mL	50.0	45.4	49.3	49.6
	50 µg/mL	63.7	56.3	62.2	61.5
	25 µg/mL	76.4	70.2	75.1	73.4
	12.5 µg/mL	92.5	82.8	87.4	86.8
	6.25 µg/mL	100	97.5	100	99.3
	3.125 µg/mL	100	100	100	100
	1.56 µg/mL	100	100	100	100
**17b**	100 µg/mL	38.4	34.8	37.8	35.6
	50 µg/mL	51.6	47.2	54.3	48.1
	25 µg/mL	62.2	60.3	69.1	60.2
	12.5 µg/mL	76.5	73.0	89.4	72.5
	6.25 µg/mL	93.1	90.6	100	93.7
	3.125 µg/mL	100	100	100	100
	1.56 µg/mL	100	100	100	100
**18a**	100 µg/mL	20.2	16.7	18.2	14.7
	50 µg/mL	32.0	24.6	26.0	23.9
	25 µg/mL	41.9	33.4	38.9	32.8
	12.5 µg/mL	55.3	45.2	51.2	46.1
	6.25 µg/mL	71.6	68.6	69.7	64.0
	3.125 µg/mL	94.3	85.5	86.2	87.4
	1.56 µg/mL	100	100	100	100
**18b**	100 µg/mL	38.5	45.7	48.3	39.3
	50 µg/mL	54.7	56.6	60.1	54.7
	25 µg/mL	67.3	71.3	72.9	70.8
	12.5 µg/mL	89.6	82.8	85.2	90.9
	6.25 µg/mL	100	96.1	98.4	100
	3.125 µg/mL	100	100	100	100
	1.56 µg/mL	100	100	100	100
**19**	100 µg/mL	25.4	24.1	28.1	26.3
	50 µg/mL	36.8	35.2	37.2	37.0
	25 µg/mL	47.9	46.0	50.4	49.2
	12.5 µg/mL	59.2	57.8	62.6	60.1
	6.25 µg/mL	77.0	70.9	83.3	75.9
	3.125 µg/mL	94.7	91.4	97.5	92.7
	1.56 µg/mL	100	100	100	100
